# ﻿Comprehensive revision of *Lycogala* (Myxomycetes) in subtropical China: morphological and phylogenetic insights and ten new species

**DOI:** 10.3897/imafungus.16.147535

**Published:** 2025-05-28

**Authors:** Wen-Long Song, Zi-Qiong Jiang, Min Li, Dmytro Leontyev, Yang Gao, Shuang-Lin Chen

**Affiliations:** 1 School of Life Sciences, Nanjing Normal University, 1 Wenyuan Road, Nanjing 210023, Jiangsu Province, China Nanjing Normal University Nanjing China; 2 College of Food Science and Pharmaceutical Engineering, Zaozhuang University, 1 Beian Road, Zaozhuang 277160, Shandong Province, China Zaozhuang University Zaozhuang China; 3 Department of Botany, H.S. Skovoroda Kharkiv National Pedagogical University, 29 Alchevskich, Kharkiv 61018, Ukraine H.S. Skovoroda Kharkiv National Pedagogical University Kharkiv Ukraine; 4 Institute of Botany and Landscape Ecology, University of Greifswald, 15 Soldmannstr, Greifswald 17487, Germany University of Greifswald Greifswald Germany; 5 College of Bioscience and Bioengineering, Jiangxi Agricultural University, 1101 Zhimin Road, Nanchang 330045, Jiangxi Province, China Jiangxi Agricultural University Nanchang China

**Keywords:** *Myxogastria*, new record to China, new taxa, species delimitation, taxonomy

## Abstract

In recent years, significant advancements have been made in the taxonomy and phylogenetics of *Lycogala*, leading to the description of numerous new species. However, *Lycogala* in China has never been systematically revised, and only eight species have been recorded. In this study, specimens of *Lycogala* from 22 sites in 11 provinces or cities of subtropical China were studied in terms of morphology, two-gene phylogenetic analysis (nuclear 18S rDNA and cytochrome oxidase subunit I), and ASAP species delimitation. We provide a checklist, which includes 21 species of *Lycogala* collected in subtropical China. These species include (1) seven species already known from the country, for which we report new localities, (2) four species, new to China (*Lycogalaalisaulianovae*, *L.fossiculatum*, *L.skovorodaense*, and *L.succineum*) and (3) ten species new to science, including *L.annulatum***sp. nov.**, *L.chinense***sp. nov.**, *L.convexum***sp. nov.**, *L.fasciculovesiculiferum***sp. nov.**, *L.helvolum***sp. nov.**, *L.indirubinum***sp. nov.**, *L.nigrum***sp. nov.**, *L.planovesiculiferum***sp. nov.**, *L.projectum***sp. nov.**, and *L.uviforme***sp. nov.** A comprehensive morphological description, detailed illustrations, molecular barcoding data, and putative position in phylogenies are provided for the newly described taxa.

## ﻿Introduction

*Myxomycetes* (*Myxogastria*) are a group of fungus-like organisms within *Amoebozoa* (Adl et al. 2019; Hyde et al. 2024), commonly found in various terrestrial ecosystems worldwide. Their life cycle consists of four main stages: spores, myxamoebae (or swarm cells), plasmodium, and fruiting bodies (Keller et al. 2022). The myxomycete genus *Lycogala* was established by Fries (1829) based on a pre-Linnaean name employed by Micheli in 1729. It is typically characterized by globose, conical, or pulvinate sporocarps, with a membranous peridium, covered with cell-like vesicles (Leontyev et al. 2023b). Different authors have assigned the genus *Lycogala* to various families, including *Arcyriaceae* (Rostafiński 1875), *Liceaceae* (Morgan 1893), *Lycogalaceae* (Lister 1894), and *Reticulariaceae* (Martin and Alexopoulos 1969), with the latter classification now being widely accepted (Leontyev et al. 2019). The placement of *Lycogala* within myxomycete orders has also been a subject of debate. It was considered within *Lamprosporales* (Lister 1894), *Lycogalales* (Macbride 1922), *Liceales* (Martin and Alexopoulos 1969), and *Cribrariales* (Lado and Eliasson 2022). With the introduction of molecular biology methods, Leontyev et al. (2019) proposed the establishment of the order *Reticulariales* and placed the family *Reticulariaceae* along with the genus *Lycogala* in this order to reflect phylogenetic relationships based on nuclear 18S rDNA (*SSU*) sequences.

Until 2022, identifying *Lycogala* species relied on morphological characteristics of sporocarps, peridium, capillitium, and spores (Martin and Alexopoulos 1969). Based on these morphological characteristics, only seven species of *Lycogala* were recognized as of 2022 (Lado 2005–2025). With the development of DNA sequencing technologies, sequences of *SSU* and cytochrome oxidase subunit I (* COI*) genes were used as molecular markers for the species delimitation and the reconstruction of phylogenetic relationships within *Lycogala* (Leontyev et al. 2023a). Molecular studies based on two gene markers (Leontyev et al. 2023a) have indicated that *Lycogalaepidendrum* (L.) Fr., the type of *Lycogala*, is a large complex taxon that may contain at least 60 biological species. Recently, 15 new species were described within the genus, based on morphological characters and phylogenetic analysis (Leontyev et al. 2023a). Subsequently, Yang et al. (2023) described *Lycogalaheterospora*. Currently, 23 species are accepted within *Lycogala* (Lado 2005–2025).

At the moment, only eight species of *Lycogala* have been recorded in China: *Lycogalaconfusum*, *Lycogalaconicum*, *L.epidendrum*, *Lycogalaexiguum*, *Lycogalaflavofuscum* (Li et al. 2008), *L.heterospora* (Yang et al. 2023), *Lycogalairregulare* (Song et al. 2024b), and *Lycogalamaculatum* (Leontyev et al. 2023a; Leontyev et al. 2023b). Among them, six, excluding *L.conicum* and *L.heterospora*, have been recorded in subtropical China. China spans a vast geographical scale, and the species diversity of *Lycogala* within its diverse ecosystems is expected to be correspondingly high (Rao and Chen 2023). Therefore, in this study, we conduct a comprehensive revision of *Lycogala* diversity in subtropical China, incorporating morphological characteristics, two molecular markers, and a distance-based species delimitation tool. We provide a checklist of 21 *Lycogala* species, including four species new to China and ten species new to science.

## ﻿Materials and methods

### ﻿Study specimens

**Figure 1.**
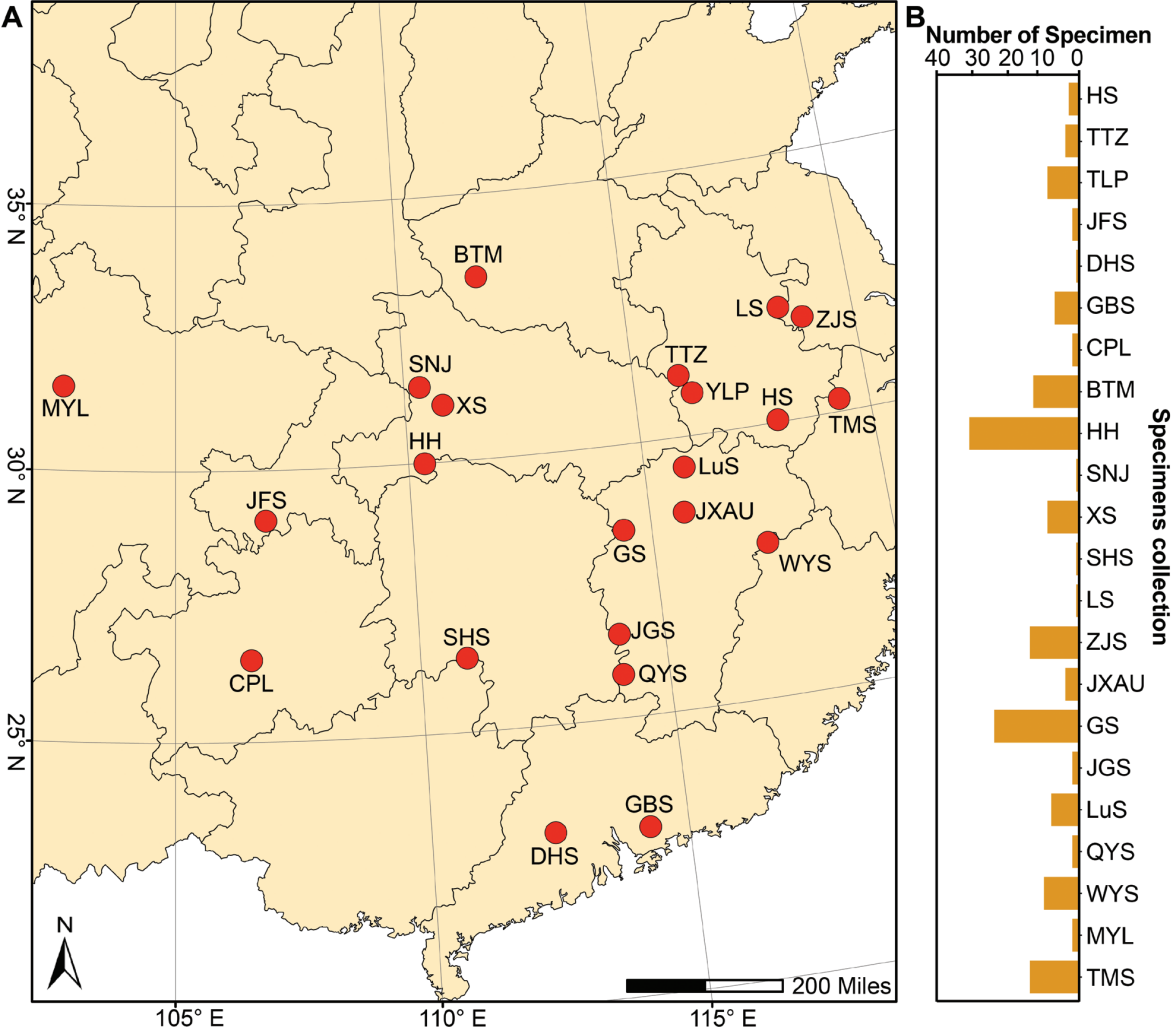
The collected site’s location of the specimens of *Lycogala* in this study (**A**) and the number of specimens collected at each location (**B**). Abbreviations: BTM, Baotianman National Nature Reserve; CPL, Changpoling National Nature Reserve; DHS, Dinghu Mountain National Nature Reserve; GBS, Gaobang Mountain National Forest Park; GS, Guan Mountain National Nature Reserve; HH, Houhe National Nature Reserve; HS, Huang Mountain National Forest Park; JFS, Jinfo Mountain National Nature Reserve; JGS, Jinggang Mountain National Nature Reserve; JXAU, Campus of Jiangxi Agricultural University; LS, Lao Mountain National Forest Park; LuS, Lu Mountain National Nature Reserve; MYL, Miyaluo Nature Reserve; QYS, Qiyun Mountain National Nature Reserve; SHS, Shunhuang Mountain National Forest Park; SNJ, Shennongjia Forestry District; YLP, Yaoluoping National Nature Reserve; TMS, Tianmu Mountain National Nature Reserve; TTZ, Tiantangzhai National Forest Park; WYS, Wuyi Mountain National Park; XS, Xingshan County; ZJS, Zijin Mountain National Forest Park.

In this study, we examined 163 specimens of *Lycogala* collected between 2008 and 2024 in 22 sites across 11 provinces and municipalities directly under the Central Government in subtropical China, including Anhui Province (16), Guangdong Province (8), Guizhou Province (2), Henan Province (12), Hubei Province (41), Hunan Province (1), Jiangsu Province (15), Jiangxi Province (50), Sichuan Province (2), Zhejiang Province (14), and Chongqing City (2) (Fig. 1, Table 1). These specimens were deposited in the Herbarium of Fungi of Nanjing Normal University (HFNNU, Nanjing, Jiangsu Province, China) and the Herbarium Mycologicum, Academiae Sinicae (HMAS, Beijing, China).

The nomenclature of myxomycete taxa mentioned in this paper follows Lado (2005–2025).

**Table 1.** The information of the specimens used for DNA extraction and sequence generation in this study.

Voucher	Species	GenBank accession No.		Geographic location	GPS coordinates (Latitude, Longitude)	Date of collection
		*SSU*	* COI*			
HFNNU 9992	*Lycogalaconicum*	PQ685963	—	Zhejiang Province: Tianmu Mountain National Nature Reserve	—	18-Jun-24
HFNNU 9993	*Lycogalaskovorodaense*	PQ685964	PQ728408	Zhejiang Province: Tianmu Mountain National Nature Reserve	30.3299°N, 119.4543°E	18-Jun-24
HFNNU 9994	*Lycogalaepidendrum*	PQ685965	PQ728409	Zhejiang Province: Tianmu Mountain National Nature Reserve	30.3346°N, 119.4499°E	18-Jun-24
HFNNU 9995	*Lycogalaalisaulianovae*	PQ685966	—	Zhejiang Province: Tianmu Mountain National Nature Reserve	30.3234°N, 119.4538°E	18-Jun-24
HFNNU 10815	*Lycogalaskovorodaense*	PQ685811	PQ728311	Jiangsu Province: Zijin Mountain National Forest Park	32.0806°N, 118.8444°E	20-Jul-24
HFNNU 10816	*Lycogalaskovorodaense*	PQ685812	PQ728312	Jiangsu Province: Zijin Mountain National Forest Park	32.0769°N, 118.8447°E	20-Jul-24
HFNNU 10817	*Lycogalafossiculatum*	PQ685813	—	Jiangsu Province: Zijin Mountain National Forest Park	32.0622°N, 118.8761°E	18-Jul-24
HFNNU 10818	*Lycogala “densum*”	PQ685814	PQ728313	Zhejiang Province: Tianmu Mountain National Nature Reserve	30.344°N, 119.4433°E	18-Jun-24
**HFNNU 10819**	***Lycogalaplanovesiculiferum***	**PQ685815**	**PQ728314**	**Zhejiang Province: Tianmu Mountain National Nature Reserve**	** 30.3233°N, 119.4562°E **	**17-Jun-24**
**HFNNU 10820**	***Lycogalaplanovesiculiferum***	**PQ685816**	**PQ728315**	**Zhejiang Province: Tianmu Mountain National Nature Reserve**	** 30.3496°N, 119.4466°E **	**18-Jun-24**
**HFNNU 10821**	***Lycogalaplanovesiculiferum***	**PQ685817**	**PQ728316**	**Zhejiang Province: Tianmu Mountain National Nature Reserve**	** 30.3469°N, 119.4411°E **	**18-Jun-24**
HFNNU 10822	*Lycogalasuccineum*	PQ685818	PQ728317	Jiangsu Province: Zijin Mountain National Forest Park	32.0587°N, 118.8678°E	20-Jul-24
HFNNU 10823	*Lycogala “microcarpum*”	PQ685819	PQ728318	Jiangsu Province: Zijin Mountain National Forest Park	32.0828°N, 118.8555°E	18-Jul-24
HFNNU 10824	*Lycogala “microcarpum*”	PQ685820	PQ728319	Jiangsu Province: Zijin Mountain National Forest Park	32.0854°N, 118.8516°E	20-Jul-24
HFNNU 10825	*Lycogala “microcarpum*”	PQ685821	PQ728320	Jiangsu Province: Zijin Mountain National Forest Park	32.0824°N, 118.8709°E	20-Jul-24
**HFNNU 10826**	***Lycogalaannulatum***	**PQ685822**	**PQ728321**	**Zhejiang Province: Tianmu Mountain National Nature Reserve**	** 30.3227°N, 119.4562°E **	**16-Jun-24**
**HFNNU 10827**	***Lycogalaannulatum***	**PQ685823**	**PQ728322**	**Zhejiang Province: Tianmu Mountain National Nature Reserve**	** 30.3463°N, 119.4456°E **	**17-Jun-24**
HFNNU 10828	*Lycogalasuccineum*	PQ685824	PQ728323	Jiangsu Province: Zijin Mountain National Forest Park	32.0623°N, 118.8748°E	18-Jul-24
**HFNNU 10829**	***Lycogalaannulatum***	**PQ685825**	**PQ728324**	**Zhejiang Province: Tianmu Mountain National Nature Reserve**	** 30.3458°N, 119.4436°E **	**18-Jun-24**
HFNNU 10830	*Lycogala “guttatum*”	PQ685826	PQ728325	Zhejiang Province: Tianmu Mountain National Nature Reserve	30.3415°N, 119.4457°E	18-Jun-24
HFNNU 10831	*Lycogala “guttatum*”	PQ685827	PQ728326	Zhejiang Province: Tianmu Mountain National Nature Reserve	30.3483°N, 119.451°E	18-Jun-24
HFNNU 10833	*Lycogalasuccineum*	PQ685828	PQ728327	Jiangsu Province: Zijin Mountain National Forest Park	32.0594°N, 118.8657°E	20-Jul-24
HFNNU 10834	*Lycogalasuccineum*	PQ685829	PQ728328	Jiangsu Province: Zijin Mountain National Forest Park	32.0572°N, 118.8693°E	20-Jul-24
**HFNNU 10835**	***Lycogalahelvolum***	**PQ685830**	—	**Zhejiang Province: Tianmu Mountain National Nature Reserve**	** 30.3439°N, 119.4481°E **	**17-Jun-24**
**HFNNU 10836**	***Lycogalahelvolum***	**PQ685831**	—	**Jiangsu Province: Zijin Mountain National Forest Park**	** 32.0868°N, 118.8596°E **	**20-Jul-24**
HFNNU 10880	*Lycogalaepidendrum*	PQ685832	PQ728329	Hubei Province: Houhe National Nature Reserve	30.0849°N, 110.5737°E	17-Oct-19
HFNNU 10881	*Lycogalaepidendrum*	PQ685833	PQ728330	Hubei Province: Houhe National Nature Reserve	30.0732°N, 110.5827°E	17-Oct-19
HFNNU 11166	*Lycogalasuccineum*	PQ685834	PQ728331	Henan Province: Baotianman National Nature Reserve	33.5129°N, 111.9432°E	23-Jun-16
HFNNU 11167	*Lycogalamaculatum*	PQ685835	PQ728332	Jiangxi Province: Guan Mountain National Nature Reserve	—	12-Sep-24
HFNNU 11168	*Lycogalamaculatum*	PQ685836	PQ728333	Jiangxi Province: Guan Mountain National Nature Reserve	—	12-Sep-24
HFNNU 11169	*Lycogalaepidendrum*	PQ685837	PQ728334	Henan Province: Baotianman National Nature Reserve	33.2027°N, 111.9262°E	26-Oct-16
HFNNU 11170	*Lycogalaepidendrum*	PQ685838	PQ728335	Henan Province: Baotianman National Nature Reserve	33.5042°N, 111.9248°E	26-Oct-16
HFNNU 11171	*Lycogalaepidendrum*	PQ685839	PQ728336	Henan Province: Baotianman National Nature Reserve	33.5046°N, 111.9325°E	26-Oct-16
HFNNU 11172	*Lycogalaepidendrum*	PQ685840	PQ728337	Henan Province: Baotianman National Nature Reserve	33.5044°N, 111.9393°E	26-Oct-16
HFNNU 11173	*Lycogalaepidendrum*	PQ685841	—	Anhui Province: Yaoluoping National Nature Reserve	30.9926°N, 116.0504°E	21-Oct-21
HFNNU 11174	*Lycogalamaculatum*	PQ685842	PQ728338	Jiangxi Province: Wuyi Mountain National Park	—	29-Jul-23
HFNNU 11175	*Lycogalamaculatum*	—	PQ728339	Jiangxi Province: Wuyi Mountain National Park	—	31-Jul-23
HFNNU 11176	*Lycogalaconicum*	PQ685843	—	Chongqing City: Jinfo Mountain National Nature Reserve	29.0449°N, 107.1467°E	14-Aug-23
HFNNU 11177	*Lycogalaconicum*	PQ685844	—	Sichuan Province: Miyaluo Nature Reserve	—	11-Aug-23
HFNNU 11178	*Lycogalaconicum*	PQ685845	—	Sichuan Province: Miyaluo Nature Reserve	—	11-Aug-23
HFNNU 11179	*Lycogalaepidendrum*	PQ685846	—	Hubei Province: Houhe National Nature Reserve	30.0887°N, 110.57°E	04-Oct-18
HFNNU 11180	*Lycogalaepidendrum*	PQ685847	—	Hubei Province: Houhe National Nature Reserve	—	04-Oct-18
HFNNU 11181	*Lycogalaepidendrum*	PQ685848	—	Hubei Province: Xingshan County	—	24-Jun-18
HFNNU 11182	*Lycogalaepidendrum*	PQ685849	—	Hubei Province: Xingshan County	—	24-Jun-18
HFNNU 11183	*Lycogalaepidendrum*	PQ685850	—	Hubei Province: Xingshan County	—	24-Jun-18
HFNNU 11184	*Lycogalaepidendrum*	PQ685851	—	Hubei Province: Xingshan County	—	24-Jun-18
HFNNU 11185	*Lycogalaepidendrum*	PQ685852	—	Anhui Province: Yaoluoping National Nature Reserve	30.9923°N, 116.0771°E	22-Oct-21
HFNNU 11186	*Lycogalaepidendrum*	PQ685853	—	Anhui Province: Yaoluoping National Nature Reserve	30.9876°N, 115.0962°E	22-Oct-21
HFNNU 11187	*Lycogalaepidendrum*	PQ685854	—	Anhui Province: Yaoluoping National Nature Reserve	30.9895°N, 116.0476°E	22-Oct-21
HFNNU 11188	*Lycogalamaculatum*	—	PQ728340	Jiangxi Province: Guan Mountain National Nature Reserve	—	24-Aug-20
HFNNU 11189	*Lycogalamaculatum*	PQ685855	PQ728341	Jiangxi Province: Guan Mountain National Nature Reserve	—	24-Aug-20
HFNNU 11190	*Lycogalafossiculatum*	PQ685856	PQ728342	Jiangxi Province: Guan Mountain National Nature Reserve	28.5208°N, 114.6127°E	13-Sep-24
HFNNU 11191	*Lycogalafossiculatum*	PQ685857	PQ728343	Jiangxi Province: Guan Mountain National Nature Reserve	28.5464°N, 114.6315°E	08-Aug-24
HFNNU 11192	*Lycogalaepidendrum*	PQ685858	PQ728344	Jiangxi Province: Guan Mountain National Nature Reserve	—	12-Sep-24
HFNNU 11193	*Lycogalamaculatum*	PQ685859	PQ728345	Jiangxi Province: Qiyun Mountain National Nature Reserve	—	02-Jul-24
HFNNU 11194	*Lycogalamaculatum*	PQ685860	PQ728346	Jiangxi Province: Qiyun Mountain National Nature Reserve	—	02-Jul-24
HFNNU 11195	*Lycogalamaculatum*	PQ685861	—	Jiangxi Province: Lu Mountain National Nature Reserve	29.5589°N, 115.976°E	16-Jul-22
HFNNU 11196	*Lycogalamaculatum*	PQ685862	—	Jiangxi Province: Lu Mountain National Nature Reserve	29.5682°N, 116.003°E	16-Jul-22
HFNNU 11197	*Lycogalaepidendrum*	PQ685863	PQ728347	Hubei Province: Houhe National Nature Reserve	—	25-Jun-19
HFNNU 11198	*Lycogalaepidendrum*	PQ685864	PQ728348	Hubei Province: Houhe National Nature Reserve	—	25-Jun-19
HFNNU 11199	*Lycogalaepidendrum*	PQ685865	—	Hubei Province: Houhe National Nature Reserve	—	25-Jun-19
HFNNU 11200	*Lycogalasuccineum*	PQ685866	PQ728349	Henan Province: Baotianman National Nature Reserve	33.5152°N, 111.9399°E	23-Jun-16
HFNNU 11201	*Lycogalaepidendrum*	PQ685867	PQ728350	Jiangxi Province: Lu Mountain National Nature Reserve	29.5586°N, 115.9695°E	16-Jul-22
HFNNU 11202	*Lycogalaepidendrum*	PQ685868	—	Hubei Province: Houhe National Nature Reserve	—	25-Jun-19
HFNNU 11203	*Lycogalaepidendrum*	PQ685869	—	Hubei Province: Houhe National Nature Reserve	30.0906°N, 110.6084°E	22-Jul-19
HFNNU 11204	*Lycogalaepidendrum*	PQ685870	—	Hubei Province: Houhe National Nature Reserve	30.0912°N, 110.6156°E	22-Jul-19
HFNNU 11205	*Lycogalaepidendrum*	PQ685871	PQ728351	Hubei Province: Houhe National Nature Reserve	—	19-Oct-19
HFNNU 11206	*Lycogalaepidendrum*	PQ685872	PQ728352	Hubei Province: Houhe National Nature Reserve	30.0906°N, 110.6084°E	19-Oct-19
HFNNU 11207	*Lycogalaepidendrum*	PQ685873	PQ728353	Hubei Province: Houhe National Nature Reserve	30.0912°N, 110.6156°E	19-Oct-19
HFNNU 11208	*Lycogalaepidendrum*	PQ685874	—	Hubei Province: Houhe National Nature Reserve	30.0842°N, 110.6053°E	19-Oct-19
HFNNU 11209	*Lycogalaepidendrum*	PQ685875	—	Hubei Province: Houhe National Nature Reserve	30.0854°N, 110.6193°E	19-Oct-19
HFNNU 11210	*Lycogalaepidendrum*	PQ685876	—	Hubei Province: Houhe National Nature Reserve	—	19-Oct-19
HFNNU 11202	*Lycogalaepidendrum*	PQ685868	—	Hubei Province: Houhe National Nature Reserve	—	25-Jun-19
HFNNU 11211	*Lycogalaepidendrum*	PQ685877	—	Hubei Province: Houhe National Nature Reserve	30.0942°N, 110.6155°E	20-Oct-19
HFNNU 11212	*Lycogalaepidendrum*	PQ685878	PQ728354	Jiangxi Province: Campus of Jiangxi Agricultural University	—	18-Jun-22
HFNNU 11213	*Lycogalaepidendrum*	PQ685879	PQ728355	Jiangxi Province: Campus of Jiangxi Agricultural University	—	18-Jun-22
HFNNU 11214	*Lycogalaepidendrum*	PQ685880	—	Hubei Province: Houhe National Nature Reserve	—	19-Oct-19
HFNNU 11215	*Lycogalaepidendrum*	PQ685881	—	Hubei Province: Houhe National Nature Reserve	—	19-Oct-19
HFNNU 11216	*Lycogalaepidendrum*	PQ685882	—	Hubei Province: Houhe National Nature Reserve	—	20-Oct-19
HFNNU 11217	*Lycogalaepidendrum*	PQ685883	PQ728356	Hubei Province: Shennongjia Forestry District	—	10-Aug-17
HFNNU 11218	*Lycogalaepidendrum*	PQ685884	PQ728357	Hubei Province: Houhe National Nature Reserve	30.0838°N, 110.631°E	18-Oct-19
HFNNU 11219	*Lycogalaepidendrum*	PQ685885	PQ728358	Jiangxi Province: Guan Mountain National Nature Reserve	—	12-Sep-24
HFNNU 11220	*Lycogalaskovorodaense*	PQ685886	PQ728359	Hubei Province: Houhe National Nature Reserve	30.0808°N, 110.5808°E	30-Aug-19
HFNNU 11221	*Lycogalaepidendrum*	PQ685887	—	Anhui Province: Huang Mountain National Forest Park	30.1723°N, 118.193°E	05-Jul-08
HFNNU 11222	*Lycogalamaculatum*	PQ685888	PQ728360	Jiangxi Province: Guan Mountain National Nature Reserve	28.5428°N, 114.6326°E	07-Aug-24
HFNNU 11223	*Lycogalaepidendrum*	PQ685889	—	Hubei Province: Houhe National Nature Reserve	—	18-Oct-19
HFNNU 11224	*Lycogalaepidendrum*	PQ685890	—	Anhui Province: Tiantangzhai National Forest Park	31.1632°N, 115.7793°E	28-Sep-16
HFNNU 11225	*Lycogalaepidendrum*	PQ685891	PQ728361	Jiangxi Province: Guan Mountain National Nature Reserve	28.5478°N, 114.6025°E	31-Aug-23
HFNNU 11226	*Lycogalaepidendrum*	PQ685892	PQ728362	Jiangxi Province: Guan Mountain National Nature Reserve	28.5496°N, 114.6086°E	01-Sep-23
HFNNU 11227	*Lycogalaepidendrum*	PQ685893	—	Hubei Province: Xingshan County	—	16-Aug-18
HFNNU 11228	*Lycogalaepidendrum*	PQ685894	—	Hubei Province: Xingshan County	—	24-Jun-18
HFNNU 11229	*Lycogalaepidendrum*	PQ685895	PQ728363	Jiangxi Province: Guan Mountain National Nature Reserve	—	12-Sep-24
HFNNU 11230	*Lycogalaepidendrum*	PQ685896	PQ728364	Jiangxi Province: Guan Mountain National Nature Reserve	—	12-Sep-24
HFNNU 11231	*Lycogalaepidendrum*	PQ685897	PQ728365	Jiangxi Province: Guan Mountain National Nature Reserve	—	12-Sep-24
HFNNU 11232	*Lycogalaconicum*	PQ685898	—	Hubei Province: Houhe National Nature Reserve	30.0783°N, 110.57°E	23-Jul-19
HFNNU 11233	*Lycogalaconicum*	PQ685899	—	Hubei Province: Houhe National Nature Reserve	30.0807°N, 110.5822°E	23-Jul-19
HFNNU 11234	*Lycogalaepidendrum*	PQ685900	PQ728366	Jiangxi Province: Lu Mountain National Nature Reserve	—	02-Aug-23
HFNNU 11235	*Lycogalaepidendrum*	PQ685901	PQ728367	Jiangxi Province: Lu Mountain National Nature Reserve	—	02-Aug-23
**HFNNU 11236**	***Lycogalachinense***	**PQ685902**	**PQ728368**	**Jiangxi Province: Guan Mountain National Nature Reserve**	** 28.5588°N, 114.5998°E **	**29-Jun-20**
**HFNNU 11237**	***Lycogalachinense***	**PQ685903**	**PQ728369**	**Jiangxi Province: Guan Mountain National Nature Reserve**	** 28.5509°N, 114.6036°E **	**01-Sep-23**
**HFNNU 11238**	***Lycogalachinense***	**PQ685904**	**PQ728370**	**Jiangxi Province: Jinggang Mountain National Nature Reserve**	** 26.6625°N, 114.0844°E **	**18-Jul-20**
**HFNNU 11239**	***Lycogalauviforme***	**PQ685905**	—	**Hubei Province: Xingshan County**	—	**24-Jun-17**
**HFNNU 11240**	***Lycogalauviforme***	**PQ685906**	—	**Jiangsu Province: Lao Mountain National Forest Park**	** 32.1191°N, 118.6341°E **	**24-Jul-15**
**HFNNU 11241**	***Lycogalauviforme***	**PQ685907**	—	**Hubei Province: Xingshan County**	—	**24-Jun-17**
**HFNNU 11242**	***Lycogalauviforme***	**PQ685908**	—	**Jiangxi Province: Lu Mountain National Nature Reserve**	** 29.5669°N, 115.9941°E **	**16-Jul-22**
**HFNNU 11243**	***Lycogalaconvexum***	**PQ685909**	**PQ728371**	**Jiangxi Province: Guan Mountain National Nature Reserve**	** 28.5213°N, 114.6069°E **	**13-Sep-24**
**HFNNU 11244**	***Lycogalaconvexum***	**PQ685910**	**PQ728372**	**Jiangxi Province: Guan Mountain National Nature Reserve**	** 28.5185°N, 114.9119°E **	**13-Sep-24**
**HFNNU 11245**	***Lycogalaconvexum***	**PQ685911**	**PQ728373**	**Jiangxi Province: Campus of Jiangxi Agricultural University**	** 28.7689°N, 115.8388°E **	**21-Oct-19**
**HFNNU 11246**	***Lycogalaconvexum***	**PQ685912**	**PQ728374**	**Jiangxi Province: Campus of Jiangxi Agricultural University**	** 28.767°N, 115.8375°E **	**14-Jul-21**
**HFNNU 11247**	***Lycogalaprojectum***	**PQ685913**	**PQ728375**	**Jiangxi Province: Jinggang Mountain National Nature Reserve**	** 26.6727°N, 114.0616°E **	**18-Jul-20**
**HFNNU 11248**	***Lycogalaprojectum***	**PQ685914**	**PQ728376**	**Anhui Province: Tiantangzhai National Forest Park**	** 31.1584°N, 115.7803°E **	**26-Jul-16**
**HFNNU 11249**	***Lycogalaprojectum***	**PQ685915**	**PQ728377**	**Anhui Province: Tiantangzhai National Forest Park**	** 31.1532°N, 115.7838°E **	**26-Jul-16**
HFNNU 11250	*Lycogalamaculatum*	PQ685916	PQ728378	Guizhou Province: Changpoling National Nature Reserve	26.6622°N, 106.6771°E	20-Aug-23
HFNNU 11251	*Lycogalamaculatum*	PQ685917	PQ728379	Guizhou Province: Changpoling National Nature Reserve	26.6658°N, 106.6879°E	20-Aug-23
HFNNU 11252	*Lycogalaepidendrum*	PQ685918	PQ728380	Jiangxi Province: Wuyi Mountain National Park	—	03-Jul-23
HFNNU 11253	*Lycogalaepidendrum*	PQ685919	PQ728381	Jiangxi Province: Wuyi Mountain National Park	—	03-Jul-23
HFNNU 11254	*Lycogalaepidendrum*	PQ685920	PQ728382	Jiangxi Province: Wuyi Mountain National Park	—	03-Jul-23
HFNNU 11255	*Lycogalaskovorodaense*	PQ685921	PQ728383	Jiangsu Province: Zijin Mountain National Forest Park	32.0831°N, 118.8402°E	20-Jul-24
HFNNU 11256	*Lycogala “densum*”	PQ685922	PQ728384	Jiangxi Province: Guan Mountain National Nature Reserve	28.5218°N, 114.6174°E	13-Sep-24
HFNNU 11257	*Lycogala “densum*”	PQ685923	PQ728385	Jiangxi Province: Wuyi Mountain National Park	—	31-Jul-23
HFNNU 11258	*Lycogala “densum*”	PQ685924	PQ728386	Jiangxi Province: Wuyi Mountain National Park	—	31-Jul-23
HFNNU 11259	*Lycogala “densum*”	PQ685925	PQ728387	Jiangxi Province: Wuyi Mountain National Park	—	29-Jul-23
HFNNU 11260	*Lycogala “densum*”	PQ685926	PQ728388	Jiangxi Province: Wuyi Mountain National Park	—	31-Jul-23
HFNNU 11261	*Lycogala “densum*”	PQ685927	—	Henan Province: Baotianman National Nature Reserve	33.5072°N, 111.9456°E	22-Jul-16
HFNNU 11262	*Lycogala “densum*”	PQ685928	—	Jiangxi Province: Wuyi Mountain National Park	—	01-Aug-23
HFNNU 11263	*Lycogalaepidendrum*	PQ685929	PQ728389	Jiangxi Province: Guan Mountain National Nature Reserve	—	12-Sep-24
HFNNU 11264	*Lycogalaepidendrum*	PQ685930	PQ728390	Jiangxi Province: Guan Mountain National Nature Reserve	—	12-Sep-24
**HFNNU 11266**	***Lycogalanigrum***	**PQ685932**	—	**Jiangsu Province: Zijin Mountain National Forest Park**	** 32.0628°N, 118.8761°E **	**10-Jun-15**
**HFNNU 11267**	***Lycogalanigrum***	**PQ685933**	—	**Anhui Province: Huang Mountain National Forest Park**	** 30.1421°N, 118.2071°E **	**05-Jul-08**
HFNNU 11268	*Lycogala “guttatum*”	PQ685934	PQ728391	Henan Province: Baotianman National Nature Reserve	33.5008°N, 111.9319°E	22-Jul-16
HFNNU 11269	*Lycogala “guttatum*”	PQ685935	PQ728392	Henan Province: Baotianman National Nature Reserve	33.504°N, 111.9459°E	23-Jun-16
**HFNNU 11270**	***Lycogalaindirubinum***	**PQ685936**	**PQ728393**	**Hubei Province: Houhe National Nature Reserve**	** 30.0893°N, 110.5666°E **	**17-Oct-19**
HFNNU 11271	*Lycogala “densum*”	PQ685937	PQ728394	Henan Province: Baotianman National Nature Reserve	33.5171°N, 111.9463°E	22-Jul-16
**HFNNU 11274**	***Lycogalafasciculovesiculiferum***	**PQ685938**	—	**Hubei Province: Houhe National Nature Reserve**	** 30.0925°N, 110.5932°E **	**20-Jul-19**
**HFNNU 11275**	***Lycogalafasciculovesiculiferum***	**PQ685939**	—	**Guangdong Province: Dinghu Mountain National Nature Reserve**	** 23.1798°N, 112.548°E **	**29-May-14**
**HFNNU 11276**	***Lycogalafasciculovesiculiferum***	**PQ685940**	—	**Hubei Province: Houhe National Nature Reserve**	** 30.0874°N, 110.6076°E **	**30-Aug-19**
**HFNNU 11277**	***Lycogalafasciculovesiculiferum***	**PQ685941**	—	**Hubei Province: Houhe National Nature Reserve**	** 30.0879°N, 110.6382°E **	**30-Aug-19**
**HFNNU 11278**	***Lycogalafasciculovesiculiferum***	**PQ685942**	—	**Hubei Province: Houhe National Nature Reserve**	** 30.0907°N, 110.621°E **	**30-Aug-19**
**HFNNU 11279**	***Lycogalafasciculovesiculiferum***	**PQ685943**	—	**Henan Province: Baotianman National Nature Reserve**	** 33.5145°N, 111.9492°E **	**25-Sep-16**
**HFNNU 11280**	***Lycogalafasciculovesiculiferum***	**PQ685944**	—	**Hunan Province: Shunhuang Mountain National Forest Park**	** 26.4037°N, 111.0553°E **	**13-Jul-15**
**HFNNU 11281**	***Lycogalafasciculovesiculiferum***	**PQ685945**	—	**Henan Province: Baotianman National Nature Reserve**	** 33.5172°N, 111.9506°E **	**25-Sep-16**
**HFNNU 11282**	***Lycogalaindirubinum***	**PQ685946**	**PQ728395**	**Chongqing City: Jinfo Mountain National Nature Reserve**	** 29.0471°N, 107.1529°E **	**14-Aug-23**
HFNNU 11283	*Lycogala “melleum*”	PQ685947	PQ728396	Anhui Province: Tiantangzhai National Forest Park	31.1593°N, 115.7735°E	26-Jul-16
HFNNU 11284	*Lycogala “melleum*”	PQ685948	PQ728397	Guangdong Province: Gaobang Mountain National Forest Park	23.0841°N, 114.3723°E	19-Jul-13
HFNNU 11285	*Lycogala “melleum*”	PQ685949	PQ728398	Guangdong Province: Gaobang Mountain National Forest Park	23.081°N, 114.3714°E	19-Jul-13
HFNNU 11286	*Lycogala “melleum*”	PQ685950	PQ728399	Jiangsu Province: Zijin Mountain National Forest Park	32.0709°N, 118.856°E	20-Jul-24
HFNNU 11287	*Lycogala “melleum*”	PQ685951	PQ728400	Guangdong Province: Gaobang Mountain National Forest Park	23.0729°N, 114.3679°E	19-Jul-13
HFNNU 11288	*Lycogala “melleum*”	PQ685952	PQ728401	Guangdong Province: Gaobang Mountain National Forest Park	23.0742°N, 114.3767°E	19-Jul-13
HFNNU 11289	*Lycogala “melleum*”	PQ685953	PQ728402	Guangdong Province: Gaobang Mountain National Forest Park	23.076°N, 114.3779°E	19-Jul-13
HFNNU 11293	*Lycogalaepidendrum*	PQ685954	—	Jiangxi Province: Lu Mountain National Nature Reserve	—	02-Aug-23
HFNNU 11320	*Lycogalaepidendrum*	PQ685955	PQ728403	Jiangxi Province: Lu Mountain National Nature Reserve	—	02-Aug-23
HFNNU 11333	*Lycogalaexiguum*	PQ685956	—	Hubei Province: Houhe National Nature Reserve	30.0814°N, 110.5744°E	26-Jun-19
HFNNU 11341	*Lycogalaepidendrum*	PQ685957	PQ728404	Jiangxi Province: Guan Mountain National Nature Reserve	—	30-Aug-23
HFNNU 11342	*Lycogalaepidendrum*	PQ685958	PQ728405	Jiangxi Province: Guan Mountain National Nature Reserve	—	30-Aug-23
HFNNU 11343	*Lycogalaepidendrum*	PQ685959	PQ728406	Jiangxi Province: Guan Mountain National Nature Reserve	—	30-Aug-23
HFNNU 11344	*Lycogala “microcarpum*”	PQ685960	—	Guangdong Province: Gaobang Mountain National Forest Park	23.0858°N, 114.3792°E	19-Jul-13
HFNNU 11345	*Lycogala “microcarpum*”	PQ685961	—	Guangdong Province: Gaobang Mountain National Forest Park	23.0862°N, 114.375°E	19-Jul-13
**HFNNU 11346**	***Lycogalaplanovesiculiferum***	**PQ685962**	**PQ728407**	**Anhui Province: Huang Mountain National Forest Park**	** 30.1573°N, 118.193°E **	**05-Jul-08**
HMAS 295544	*Lycogalaepidendrum*	PQ685968	—	Anhui Province: Yaoluoping National Nature Reserve	30.9854°N, 116.0311°E	21-Oct-21
HMAS 295547	*Lycogalaepidendrum*	PQ685967	—	Anhui Province: Yaoluoping National Nature Reserve	30.9851°N, 116.0318°E	21-Oct-21
HMAS 295549	*Lycogalaepidendrum*	PQ685969	—	Anhui Province: Yaoluoping National Nature Reserve	30.984°N, 116.0311°E	22-Oct-21
HMAS 295566	*Lycogalaepidendrum*	PQ685970	—	Anhui Province: Yaoluoping National Nature Reserve	30.9864°N, 116.0926°E	22-Oct-21
HMAS 295569	*Lycogalaepidendrum*	PQ685971	—	Anhui Province: Yaoluoping National Nature Reserve	30.9857°N, 116.0939°E	22-Oct-21

Note: The symbol ‘—’ indicates that data is unavailable. The substrate of all specimens is rotten wood. Samples representing new species are in bold. Abbreviations: COI: cytochrome oxidase subunit I; HFNNU: Herbarium of Fungi of Nanjing Normal University; HMAS: Herbarium Mycologicum, Academiae Sinicae; SSU: nuclear 18S rDNA.

### ﻿Morphological studies

Sporocarps were observed and measured using a JSZ6 dissecting microscope (Jiangnan Yongxin Optics Co., Jiangsu Province, China). Microscope slides with peridium were prepared using Hoyer’s mounting medium (Phygene Biotech, Fujian Province, China). Microscope slides with capillitium and spores were prepared using the lactophenol cotton blue stain solution (Beijing Solarbio Science & Technology Co., Ltd., Beijing, China). Observations and measurements of microscopic characteristics were performed with an Axio Imager A1 light microscope (Carl Zeiss AG, Göttingen, Germany). The diameters of 10 peridium vesicles were determined at a magnification of 20×. The diameters of 10 capillitium threads and 50 spores of each specimen were determined with an oil immersion objective at a magnification of 100× (Song and Chen 2024). Spores were measured with ornamentation. The range of variation diameter for capillitium and spores is given in descriptions as rounded (minimum–)25^th^ percentile–75^th^ percentile (–maximum) (Leontyev et al. 2023b). For SEM observations, air-dried specimens were mounted on aluminum stubs with adhesive membranes, sputtered with gold using a GVC-2000 ion sputtering tool (Gewei Instrument Co., Beijing, China) and observed under a Phenom XL G2 scanning electron microscope (Phenom Scientific, Netherlands) at 15 kV (Song et al. 2024a).

All descriptions of new species in this paper strictly follow the rules set by Schnittler et al. (2025).

### ﻿DNA extraction, PCR amplification, and sequencing

Approximately 10 mg of the fruiting body from each specimen was picked up with forceps under a dissecting microscope and placed in a sterile 1.5 mL Eppendorf tube containing three glass beads with a diameter of 2.5 mm. DNA extraction followed the protocol of the DP316 TIANamp Micro DNA Kit (Tiangen Biotech (Beijing) Co., Ltd., Beijing, China).

To reconstruct the phylogeny of *Lycogala*, two independent genetic markers (*SSU* and * COI*) were obtained by Sanger sequencing. Approximately 550 base pairs (bp) from the 5′ end of *SSU*, a fragment that is free of introns, were amplified with the primers S1a (5’–3’: CTGGTTGATCCTGCCAGAAT) and R_SIP (5’–3’: GCACCAGACTTGTCCTCC) (Dagamac et al. 2017; Leontyev et al. 2023a). The ~520 bp of the * COI* were amplified with the primers COMF2 (5’–3’: GCACCTGATATGGCKTTTC) and COIR2 (5’–3’: ACGTCCATACCTACTTATAC) (Leontyev et al. 2023a). The polymerase chain reaction (PCR) protocol for *SSU* was as follows: initial denaturation at 98 °C for 2 min, 30 cycles of denaturation at 98 °C for 10 s, annealing at 56 °C for 10 s, and elongation at 72 °C for 15 s, followed by the final elongation at 72 °C for 5 min. For * COI*, the PCR protocol was somewhat different: 98 °C for 2 min, 30 cycles of denaturation at 98 °C for 10 s, annealing at 52 °C for 10 s, and elongation at 72 °C for 15 s, followed by the final elongation at 72 °C for 5 min. The PCR mixture contained 25 μL 2X T8 High-Fidelity Master Mix (Tsingke Biotech, Beijing, China), 1 μL of each primer, forward and reverse (10 mM), 1 μL template DNA, and completed with water up to a final volume of 50 μL. All PCR products were sequenced by Sangon Biotech (Shanghai) Co., Ltd. (Shanghai, China).

### ﻿Sequence alignment and phylogenetic analyses

A comprehensive search of the NCBI GenBank database was conducted to retrieve *SSU* and * COI* sequences associated with species of the genus *Lycogala*. The final dataset consisted of a total of 423 sequences, including 260 (161 *SSU* and 99 * COI*) newly obtained sequences from 163 specimens (Table 1) and 305 sequences (247 *SSU* and 58 * COI*) retrieved from GenBank (Suppl. materials 1, 2, 3).

*SSU* and * COI* sequences were aligned using MAFFT ver. 7.471 (Katoh and Standley 2013) with the E-INS-i option for *SSU* and the G-INS-i option for * COI* with default gap penalties (Song et al. 2024b) in PhyloSuite ver. 1.2.3 (Zhang et al. 2020; Xiang et al. 2023). Ambiguously aligned fragments, primer binding sites, gaps, and low-quality ends were removed using Gblocks (Talavera and Castresana 2007) with the following parameter settings: minimum number of sequences for a conserved/flank position (208/208 for *SSU* and 81/81 for * COI*), maximum number of contiguous non-conserved positions (8), minimum length of a block (10), allowed gap positions (all). A two-gene phylogeny was built from a concatenated dataset with the following partitions: *SSU* 1–609, * COI* 610–1218 (Suppl. materials 4, 5). The best-fit model of nucleotide substitution was determined by ModelFinder (Kalyaanamoorthy et al. 2017) for each partition using the corrected Akaike Information Criterion (AICc) criterion: JC+G4 for *SSU* and TPM2+F+I+G4 for * COI*. Maximum likelihood (ML) analyses were conducted with IQ-TREE ver. 1.6.8 (Minh et al. 2013; Nguyen et al. 2015). The ultrafast bootstraps (UBS) with 1000 pseudo replicates and Bayesian posterior probabilities (PP) were performed to evaluate the statistical support of clades. The best fitting substitution models for Bayesian inference (BI) analysis were as follows: JC+G4 for *SSU* and GTR+F+I+G4 for * COI*. BI analyses were conducted using MrBayes ver. 3.2.6 (Ronquist et al. 2012), with four separate chains each 10 × 10^6^ generations long (sampling every 1000^th^ generation). The convergence of Markov Chain Monte Carlo (MCMC) simulations was estimated using Tracer 1.7.2 (Rambaut et al. 2018) and by the average standard deviation of split frequencies. Consequently, the first 19% of sampled data and trees were discarded as burn-in. ML and BI trees were visualized in FigTree ver. 1.4.4 (Rambaut 2018) and edited with Adobe Illustrator 2021 (San Jose, CA, USA). Members of the genera *Reticularia* and *Enteridium* were used to root the trees.

### ﻿Molecular species delimitation analysis

Assemble Species by Automatic Partitioning (ASAP), the distance-based species delimitation tool, was employed to assess putative taxonomic boundaries within *Lycogala* (Puillandre et al. 2021). The ASAP analysis was conducted on the web server (https://bioinfo.mnhn.fr/abi/public/asap/) with a Kimura K2 parameter based on the alignments of *SSU* sequences (Suppl. material 2), as described in Song et al. (2024a).

Grammarly ver. 1.115.0.0 was used during the revision process under human supervision, and its scope of application is strictly limited to correcting typing errors, standardizing punctuation, and checking grammar. No generative artificial intelligence tools were used in this study.

## ﻿Results

### ﻿Phylogenetic analysis and species delimitation

**Figure 2.**
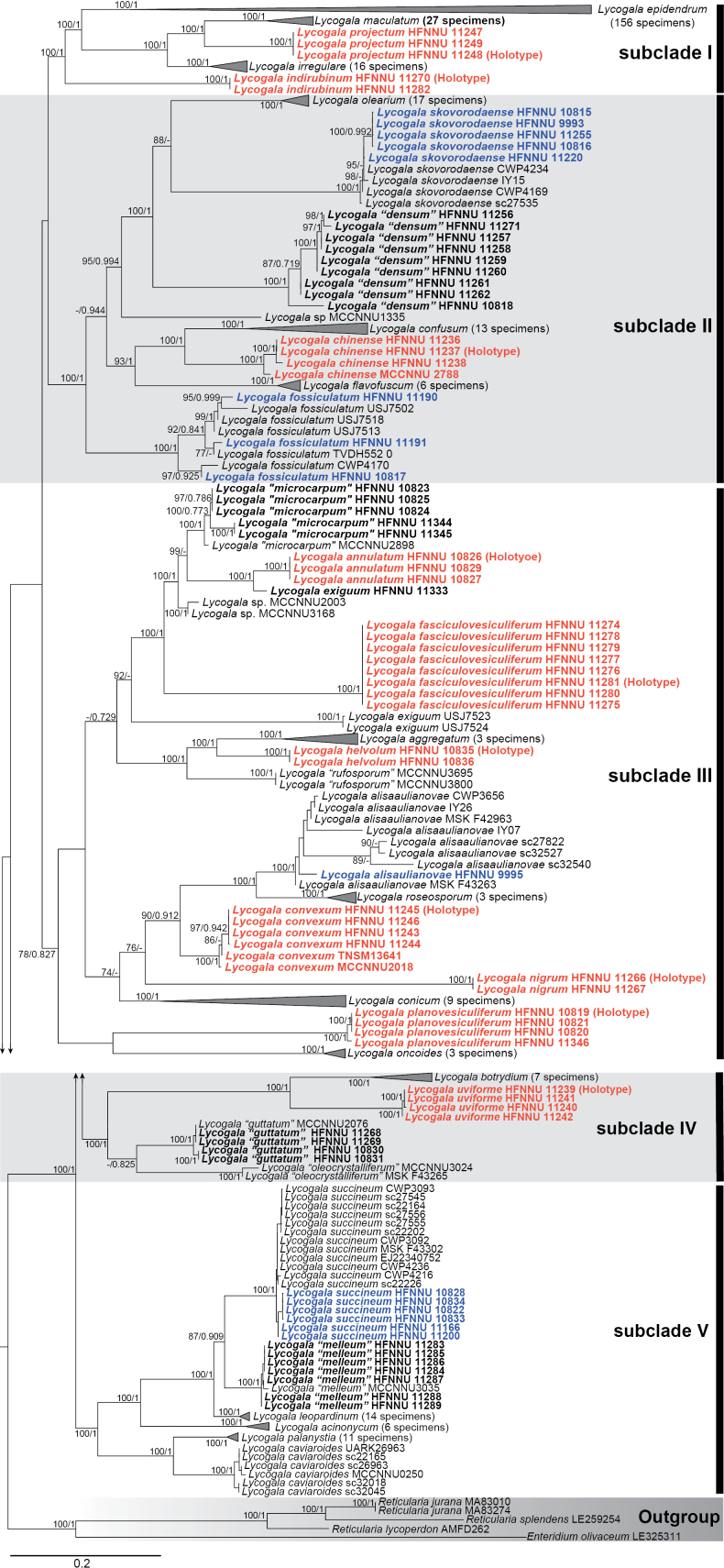
Maximum likelihood tree inferred from the concatenated sequences of *SSU* and * COI*, showing the phylogenetic relationship of *Lycogala*. The members of *Reticularia* were used to root the tree. Only Maximum likelihood ultrafast bootstrap support (UBS)/Bayesian posterior probabilities (PP) ≥ 70/0.7 are shown. The voucher numbers are indicated after the species names. The number of identical genotypes is in parentheses (if present). The ten new species described in this study are indicated in red and bold. The four new species to China are indicated in blue and bold. Other newly obtained sequences are in bold. The scale bar represents the number of substitutions per site.

In the two-gene phylogenetic tree, based on a concatenated alignment of *SSU* and * COI* sequences (Fig. 2, Suppl. material 6), all specimens of *Lycogala* cluster into a monophyletic clade (UBS = 100, PP = 1), thus supporting earlier conclusions about the monophyly of this genus (Leontyev et al. 2023a). The *Lycogala* clade branches into five subclades (Fig. 2).

The ASAP results (Suppl. material 7) indicate that the genetic distance between species of *Lycogala*, as measured from partial *SSU* sequences, ranges from 0 to 0.62, with the most common values between 0.37 and 0.41 (Suppl. materials 7, 8). According to the delimitation results, there may be 32–85 species within studied collection of *Lycogala* (Suppl. material 7).

### ﻿Checklist, description, and notes on *Lycogala* species in subtropical China

The molecular barcoding and ASAP analysis allowed to group 163 *Lycogala* specimens collected in subtropical China into 11 previously described and 13 undescribed species. Among the latter, we describe here ten new species of the genus *Lycogala*. Among the previously described species, seven had already been recorded in the country, though previously known from fewer localities, and four species (*Lycogalaalisaulianovae*, *Lycogalafossiculatum*, *Lycogalaskovorodaense*, and *Lycogalasuccineum*) are newly recorded in China. Below, we provide an alphabetically arranged checklist of *Lycogala* species found in subtropical China.

#### ﻿ Lycogala alisaulianovae

Taxon classificationFungiLicealesTubiferaceae

Leontyev, Ishchenko, Schnittler & Sarzhevskyi, in Leontyev, Ishchenko & Schnittler, Mycologia 115(4):527 (2023)

BC3FAACE-A9DB-5FFF-9134-3F7361C6EFDF

MycoBank No: 848145

Fig. 3

##### Specimen examined.

CHINA • Zhejiang Province: Tianmu Mountain National Nature Reserve, 30.3234°N, 119.4538°E, on rotten wood, 18 Jun 2014, collected by Wen-Long Song and Ya-Jing Chen (HFNNU 9995).

##### Distribution.

Ukraine, Russia, Germany (Leontyev et al. 2023b), Norway (Johannesen and Vetlesen 2024), and China (this study).

##### Notes.

**Figure 3.**
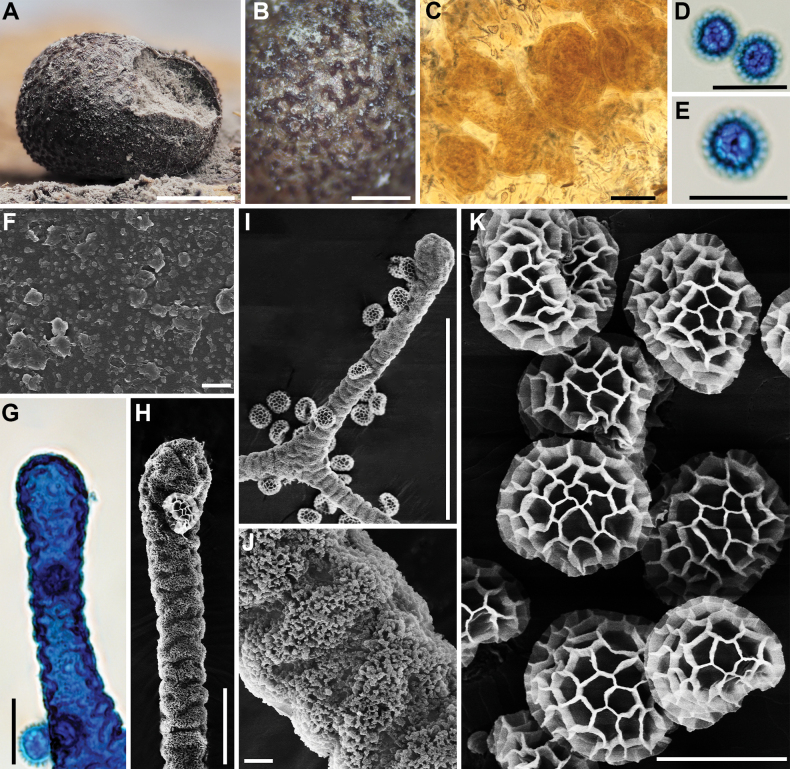
Morphological characteristics of *Lycogalaalisaulianovae*: **A** sporocarp; **B** peridium; **C** peridial vesicles; **D, E** spores; **F** inner surface of the peridium (SEM); **G** the bulbous free ends of the capillitium; **H** the bulbous free ends of the capillitium (SEM); **I** the bulbous free ends and branch of the capillitium (SEM); **J** the detail of capillitium ornamentation (SEM); **K** spores (SEM). Scale bars: 2 mm (**A**); 1 mm (**B**); 50 μm (**C, I**); 10 μm (**D, E, G, H**); 5 μm (**K**); 1 μm (**F, J**).

*Lycogalaalisaulianovae* is recorded in China for the first time. The main characteristics of this species are copper or umber brown sporocarps, solitary peridial vesicles that appear dark brown under TL, and bluish-gray spore mass, which makes it easy to identify (Leontyev et al. 2023b). Our specimen HFNNU 9995 shows slight differences from the original description (Leontyev et al. 2023b) in terms of the color of the sporocarp (brown to nearly black) and the pigmentation of the vesicles (brownish yellow), but the capillitium, spore mass color, and spore ornamentation (Fig. 3) are the same as those of the original description. The two-gene phylogenetic analysis (Fig. 2) clustered our specimen HFNNU 9995 within the branch of *L.alisaulianovae* (CWP3656, IY07, IY26, sc27822, sc32540, and sc32527) with high statistical support (UBS = 100, PP = 1), thus confirming its morphological identification as *L.alisaulianovae*.

#### ﻿ Lycogala annulatum

Taxon classificationFungiLicealesTubiferaceae

W. L. Song, Z. Q. Jiang & Shuang L. Chen sp. nov.

3A764E7E-413E-5C36-949F-5C3A8D27C8A1

MycoBank No: 857345

Fig. 4

##### GenBank accession numbers.

PQ685822 (*SSU*) and PQ728321 (* COI*).

##### Etymology.

*Annulus* (Latin) ring, referring to the ring-shaped ornamentation of the capillitium.

##### Diagnosis.

Differs from *L.exiguum* by smaller vesicle diameter, and by capillitium tubules decorated by conspicuous visible.

##### Description.

Sporocarps scattered, spherical to short horizontally ovoid, sometimes deformed by mutual pressure, 1.6–3.7 mm in diameter. Peridium membranous, thin, yellowish brown with olivaceous tones or somewhat darker, with dense vesicles cover on the outer surface; the dehiscence area at the top of sporocarp conspicuous, free of vesicles, irregularly in shape. Inner surface of the peridium smooth or covered by scattered warts. Vesicles polygonal, irregularly ovoid, black under RL, dark brown under TL, (17–)18–23(–26) μm in diameter, clustered together forming large aggregates with 2–6 vesicles across the group. Vesicles walls smooth, single-layered. Oil droplets large or small, present in the central part of each vesicle. Capillitium tubular, hyaline under TL, with wavy contour and no bracelet-like thickening, (2.1–)3.2–5.3(–7.6) μm in diameter; the surface of the thread is densely covered by small warts and large rings, the latter visible both under TL and SEM. Spore mass in old collections yellow with ochraceous undertones or darker, hyaline under TL, (5.2–)5.5–5.8(–6.2) μm in diameter, reticulate, with 6–7 meshes across diameter, unornamented area occupies 1/4 of the spore surface. Plasmodium unknown.

##### Distribution.

Currently known only from China.

##### Habitat.

On rotten wood.

##### Holotype.

CHINA • Zhejiang Province: Tianmu Mountain National Nature Reserve, 30.3227°N, 119.4562°E, on rotten wood, 16 Jun 2024, collected by Wen-Long Song and Ya-Jing Chen (HFNNU 10826).

##### Additional specimens examined.

CHINA • Zhejiang Province: Tianmu Mountain National Nature Reserve, 30.3463°N, 119.4456°E, on rotten wood, 17 Jun 2024, collected by Wen-Long Song and Ya-Jing Chen (HFNNU 10827); • Zhejiang Province: Tianmu Mountain National Nature Reserve, 30.3458°N, 119.4436°E, on rotten wood, 18 Jun 2024, collected by Wen-Long Song and Ya-Jing Chen (HFNNU 10829).

##### Notes.

The most prominent morphological characteristic of *L.annulatum* is the surface of the capillitium tubules, which is decorated with large rings visible under TL and SEM. Similar ornamentation of the capillitium is also known in *Lycogalaaggregatum* and *Lycogalabotrydium*, but these two species have different morphologies of peridial vesicles (Leontyev et al. 2023b): irregularly grouped and clustered in smooth aggregates with individual vesicles nearly indistinguishable, respectively. The structure of the peridium makes *L.annulatum* a member of the *L.exiguum* complex. However, *L.annulatum* differs from the type material of *L.exiguum* (Leontyev and Schnittler 2023) by the following morphological differences: (i) sporocarps of *L.exiguum* form very large groups (>50), while those of *L.annulatum* is scattered; (ii) the vesicle diameter of *L.exiguum* is 20–40 μm, while that of *L.annulatum* is (17–)18–23(–26) μm; (iii) the capillitium of *L.exiguum* is ornamented with pits, warts and only scanty rings 1–3 μm in diameter, while in *L.annulatum* the tubules are richly decorated with rings visible under TL. From a phylogenetic perspective based on *SSU* and * COI* sequences (Fig. 2), the three specimens representing *L.annulatum* form a sister clade with *L.exiguum*HFNNU 11333, with strong statistical supported (UBS = 100, PP = 1). The separation of *L.annulatum* as a distinct species is supported in 5 of 10 partitions identified by ASAP (Suppl. material 7). The genetic distance between *L.annulatum* and *L.exiguum*HFNNU 11333, measured based on *SSU* sequences, is 0.03, which is rather low (Leontyev et al. 2015; Borg Dahl et al. 2018). Therefore, although we describe *L.annulatum* on the basis of morphology data, the species boundaries within the *L.exiguum* complex require further investigation.

**Figure 4.**
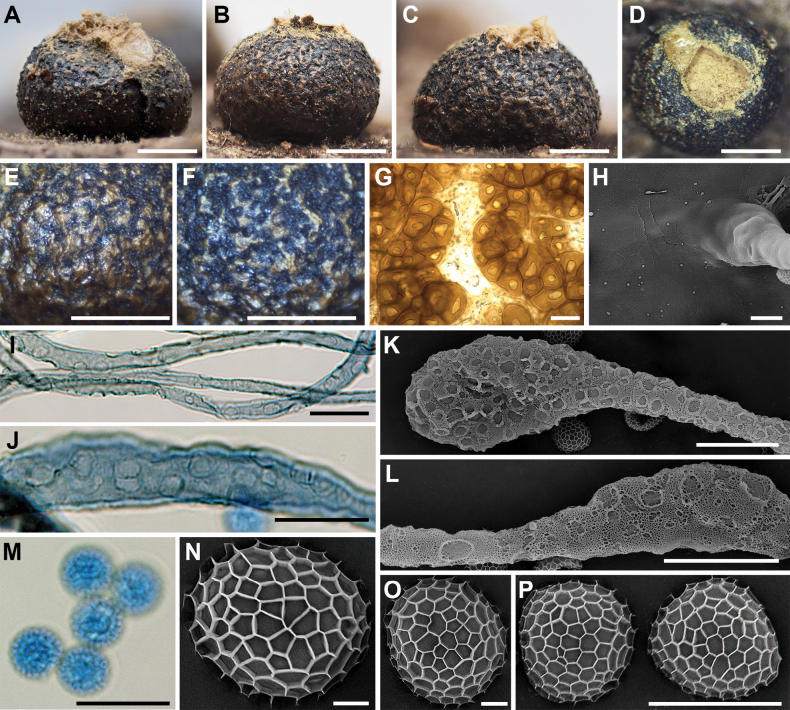
Morphological characteristics of *Lycogalaannulatum*: **A–C** sporocarp; **D** sporocarp with cracked top; **E, F** the outer surface of the peridium; **G** peridial vesicles; **H** the inner surface of the peridium (SEM); **I, J** capillitium; **K, L** the detail of capillitium ornamentation (SEM); **M** spores; **N–P** spore (SEM). Scale bars: 1 mm (**A–F**); 100 μm (**G**); 5 μm (**H, P**); 10 μm (**I–M**); 1 μm (**N, O**).

#### ﻿ Lycogala caviaroides

Taxon classificationFungiLicealesTubiferaceae

Leontyev, Schnittler, S.L. Stephenson & G. Konstantinides, in Leontyev, Ishchenko & Schnittler, Mycologia 115(4):533 (2023)

68AE25D1-56A2-5ABE-B23E-6B30037B9E3C

MycoBank No: 848149

##### Specimen examined.

NA.

##### Distribution.

Germany, Greece, United States (Leontyev et al. 2023b), and China (this study).

##### Notes.

*Lycogalacaviaroides* was separated from the *L.epidendrum* complex based on morphological characteristics and two-gene phylogenetic analysis (*SSU* and * COI*) by Leontyev et al. (2023a, b). When retrieving GenBank data for our study, we found a *COI* sequence of specimen MCCNNU0250, which was previously submitted to GenBank. This specimen originates from Tianmu Mountain National Nature Reserve, Zhejiang Province, subtropical China. Based on the * COI* sequences similarity, specimen MCCNNU0250 can be attributed to *L.caviaroides* (Fig. 2). However, we could not obtain the specimen MCCNNU0250 for the morphological study. Therefore, we cannot reliably confirm the occurrence of *L.caviaroides* in subtropical China at this time.

#### ﻿ Lycogala chinense

Taxon classificationFungiLicealesTubiferaceae

W. L. Song, Yang Gao, Leontyev & Shuang L. Chen sp. nov.

FEA06B83-C046-5261-9089-EC1AAD4ACC00

MycoBank No: 857338

Fig. 5

##### GenBank accession numbers.

PQ685903 (*SSU*) and PQ728369 (* COI*).

##### Etymology.

*Chinense* (Latin) Chinese, referring to the geographic origin of collections.

##### Diagnosis.

Differs from *L.confusum* by dark, thick-walled peridial vesicles, nearly solitary or clustered in small rows and bunches, containing large oil droplets and occasional crystals.

##### Description.

Sporocarps scattered, spherical, short horizontally oval, or somewhat irregular, 1.0–3.5 mm in diameter. Peridium thin, membranous, light ochre to yellowish brown with olivaceous tones, densely covered with peridial vesicles. The inner surface of the peridium almost smooth, with scattered patches of wart, as seen in SEM. Peridial vesicles appear brown under RL, warm pale yellow to deep warm brown under TL, (45–)90–110(–165) μm in diameter, nearly solitary or clustered by 2–4, forming rows and bunch-like groups, somewhat deformed from mutual pressure. Crystals in vesicles occasionally present in the form of druses. Oil droplets present, large, often occupy half or more of the inner vesicle space. Capillitium with wavy and bracelet-like thickenings nearly invisible under TL, but more or less conspicuous under SEM, (5.0–)8.0–12(–22.1) μm in diameter, densely ornamented by pits and warts; capillitial free ends without conspicuous swellings. Spore mass in old collections yellow with ochraceous undertones or darker, hyaline under TL, (5.5–)6.5–7.0 μm in diameter, reticulate, with 4–5 meshes across diameter, unornamented area occupies 1/4–1/3 of the spore surface. Plasmodium unknown.

##### Distribution.

Currently known only from China.

##### Habitat.

On rotten wood.

##### Holotype.

CHINA • Jiangxi Province: Guan Mountain National Nature Reserve, on rotten wood, 28.5509°N, 114.6036°E, 29 Jun 2020, collected by Xiao-Dong Liu and Yang Gao (HFNNU 11237).

##### Additional specimens examined.

CHINA • Henan Province: Baotianman National Nature Reserve, on rotten wood, 33.5190°N, 111.9470°E, 22 Jul 2016, collected by Yang Gao and Gao-Wei Wang (MCCNNU 2788); • Jiangxi Province: Guan Mountain National Nature Reserve, 28.5588°N, 114.5998°E, on rotten wood, 29 Jun 2020, collected by Xiao-Dong Liu and Yang Gao (HFNNU 11236); • Jinggang Mountain National Nature Reserve, 26.6625°N, 114.0844°E, on rotten wood, 18 Jul 2020, collected by Xiao-Dong Liu and Yang Gao (HFNNU 11238).

##### Notes.

According to our two-gene phylogeny (Fig. 2), this species is most closely related to *L.confusum*. Indeed, these two taxa share many similarities in peridial structure. In both species, the vesicles are filled with oil droplets, including relatively large ones, and form small rows or bunches, although in *L.chinense* the vesicles are often nearly solitary. However, the macromorphology of these two species differs noticeably. In *L.chinense*, the vesicles are dark brown and relatively large (up to 160 µm), situated against the light yellowish background of exposed peridium, forming a contrasting, speckled pattern. In contrast, *L.confusum* has smaller (up to 90 µm) and lighter vesicles, densely covering the orange-brown background, creating a “marbled” pattern (Leontyev et al. 2023b). *L.chinense* also resembles *L.skovorodaense* but differs in having much looser clustering of vesicles. Compared to both similar taxa, *L.confusum* and *L.skovorodaense*, the new species also differs by the occasional presence of crystals in peridial vesicles.

**Figure 5.**
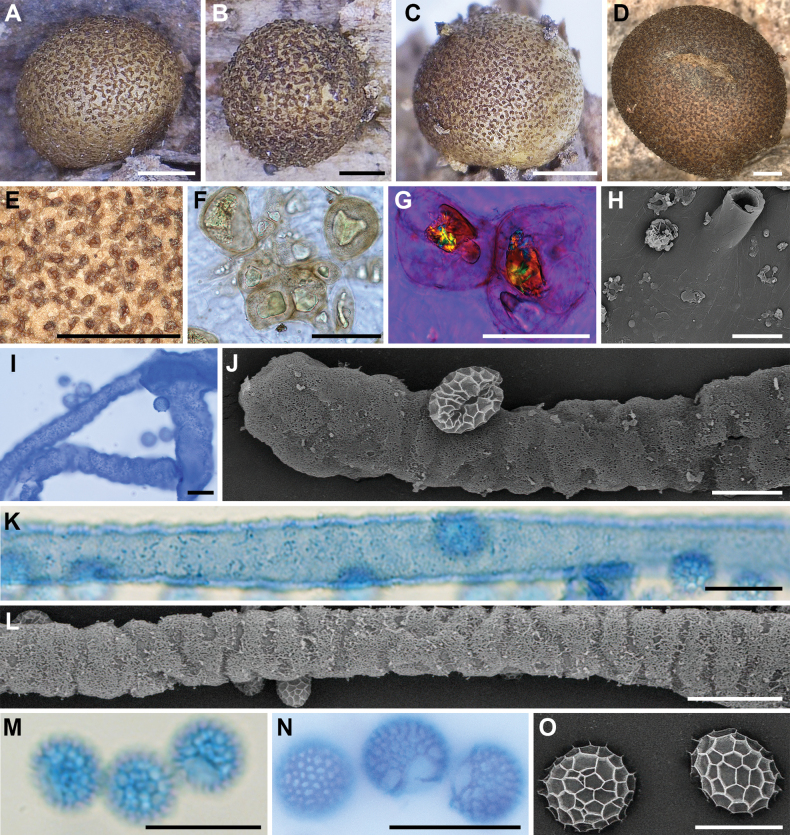
Morphological characteristics of *Lycogalachinense*: **A–D** sporocarp; **E** the out surface of the peridium; **F, G** peridial vesicles; **H** the inner surface of the peridium (SEM); **I** capillitium; **J** the bulbous free ends of capillitium (SEM); **K** capillitium; **L** the detail of capillitium ornamentation (SEM); **M, N** spores; **O** spores (SEM). Scale bars: 1 mm (**F**); 0.5 mm (**A–D**); 0.2 mm (**D**); 100 μm (**G, K–N**); 10 μm (**H, I**); 5 μm (**J, O**).

#### ﻿ Lycogala conicum

Taxon classificationFungiLicealesTubiferaceae

Pers., Syn. meth. fung. 1:159 (1801)

8B2BC157-11E4-5FB6-B37B-138D1A7618AC

MycoBank No: 356824

##### Specimen examined.

CHINA • Zhejiang Province: Tianmu Mountain National Reserve, on rotten wood, 18 Jun 2014, collected by Wen-Long Song and Ya-Jing Chen (HFNNU 9992); • Chongqing City: Jinfo Mountain National Nature Reserve, 29.0449°N, 107.1467°E, on rotten wood, 14 Aug 2023, collected by Shu-Zhen Yan and Shuang-Lin Chen (HFNNU 11176); • Sichuan Province: Miyaluo Nature Reserve, on rotten wood, 11 Aug 2023, collected by Shu-Zhen Yan and Shuang-Lin Chen (HFNNU 11177); • Sichuan Province: Miyaluo Nature Reserve, on rotten wood, 11 Aug 2023, collected by Shu-Zhen Yan and Shuang-Lin Chen (HFNNU 11178); • Hubei Province: Houhe National Nature Reserve, 30.0783°N, 110.5700°E, on rotten wood, 23 Jul 2019, collected by Min Li (HFNNU 11232); • Hubei Province: Houhe National Nature Reserve, 30.0807°N, 110.5822°E, on rotten wood, 23 Jul 2019, collected by Min Li (HFNNU 11233).

##### Distribution.

Widely distributed worldwide.

##### Notes.

*Lycogalaconicum* is a common and morphologically distinct species characterized by cone-shaped sporocarps. In this study, we found six specimens that are similar to the description of *L.conicum* Pers. (Martin and Alexopoulos 1969). Published sequences assigned to *L.conicum* form two branches (Fig. 2), one containing the specimen IY39, collected in Russia, and the other, which includes specimens EJ18190388 (Norway) and TVDH551 (Australia). Our collections cluster with the first branch (UBS = 100, PP = 1, distance of 0–0.003) and demonstrate considerable genetic distance from the second (distances 0.16 and 0.17, see Suppl. material 8). The ASAP data indicate that these two clusters of *L.conicum* may represent different species. Therefore, further research is necessary, integrating both morphological and phylogenetic data to confirm or refute the hypothesis that *L.conicum* represents a species complex.

#### ﻿ Lycogala convexum

Taxon classificationFungiLicealesTubiferaceae

W. L. Song, Yang Gao, Leontyev & Shuang L. Chen sp. nov.

9D469C2E-97E6-58E1-AFD1-F0C9AAD9EB9F

MycoBank No: 857344

Fig. 6

##### GenBank accession numbers.

PQ685911 (*SSU*) and PQ728373 (* COI*).

##### Etymology.

*Convexum* (Latin) convex, referring to the shape of peridial vesicles.

##### Diagnosis.

Differs from *L.maculatum* by convex, reddish-brown vesicles and the absence of bracelet-like ornamentation of the capillitium.

##### Description.

Sporocarps scattered or in small groups, more or less spherical, 1.5–3.5 mm in diameter. Peridium thin, membranous, pale beige-yellow, looking dark brown because of dense vesicle cover on the outer surface. The inner surface of the peridium smooth or covered with sparse warts. Vesicles under RL look like dried droplets, noticeably convex, with flattened periphery, rounded or slightly angular, wine red to rust red; under TL vesicles solitary, deep warm brown or darker t, semi-transparent, (120–)150–205(–280) μm in diameter. Vesicle walls thick, 5–15 µm, granular, single- or multilayered, with indistinct layer boundaries. Crystals mostly absent, but may be present and rather large. Oil droplets present, small to rather large, irregular in shape, strongly birefringent. Capillitium (2.1–)4.2–6.9(–10.8) μm in diameter, with uneven, wavy contour, but no clear bracelet-like thickenings, free end of tubules are swollen. The surface of capillitium ornamented with small warts and pits, accompanied by irregular ring-like inlets. Spore mass yellow with ochraceous undertones or darker in old collections, hyaline under TL, (5.0–)6.5–7.0(–7.5) μm in diameter, reticulate, with 4–5 meshes across diameter, unornamented area occupies 1/4–1/2 of the spore surface. Plasmodium unknown.

##### Distribution.

Currently known from China and South Korea.

##### Habitat.

On rotten wood.

##### Holotype.

CHINA • Jiangxi Province: Campus of Jiangxi Agricultural University, 28.7689°N, 115.8388°E, on rotten wood, 21 Oct 2019, collected by Yang Gao (HFNNU 11245).

##### Additional specimens examined.

CHINA • Jiangxi Province: Campus of Jiangxi Agricultural University, 28.7670°N, 115.8375°E, on rotten wood, 14 Jul 2021, collected by Yang Gao (HFNNU 11246); • Jiangxi Province: Guan Mountain National Nature Reserve, 28.5213°N, 114.6069°E, on rotten wood, 13 Sep 2024, collected by Xiao-Dong Liu and Yang Gao (HFNNU 11243); • Jiangxi Province: Guan Mountain National Nature Reserve, 25.5185°N, 114.9119°E, on rotten wood, 13 Sep 2024, collected by Xiao-Dong Liu and Yang Gao (HFNNU 11244); • Hubei Province: Shennongjia Forestry District, on rotten wood, 20 Aug 2015, collected by Shu-Zhen Yan, Gang He, Yang Gao, Xi Lin, and Gao-Wei Wang (MCCNNU2018). SOUTH KOREA • South Gyeongsang: Forest on Jiri Mt, 35.336°N, 127.73°E, on rotten wood, 16 Jul 2007, collector unknown (TNSM13641).

##### Notes.

*Lycogalaconvexum* resembles *L.maculatum*, although these species are not closely related. Both taxa have relatively large sporocarps, densely covered by dark solitary vesicles. However, in *L.maculatum*, both peridium and vesicles are usually darker than in *L.convexum*. Vesicles in *L.maculatum* are nearly flat, while in the new species they are clearly convex. Finally, *L.convexum* does not have bracelet-like thickenings on the capillitium, which are typical of *L.maculatum* and related *Lycogalaprojectum* (see below). From a phylogenetic perspective based on *SSU* and * COI* sequences (Fig. 2), the six specimens representing *L.convexum* form a separate clade (UBS = 100, PP = 1), sister to *L.alisaulianovae* and *Lycogalaroseoporum* (UBS = 90, PP = 0.912). Both related species share with *L.convexum* the general type of the vesicle cover, but differ by the color of the spore mass. The interspecific genetic distance from *L.convexum* to *L.roseoporum* is 0.19, and to *L.alisaulianovae* is 0.25.

**Figure 6.**
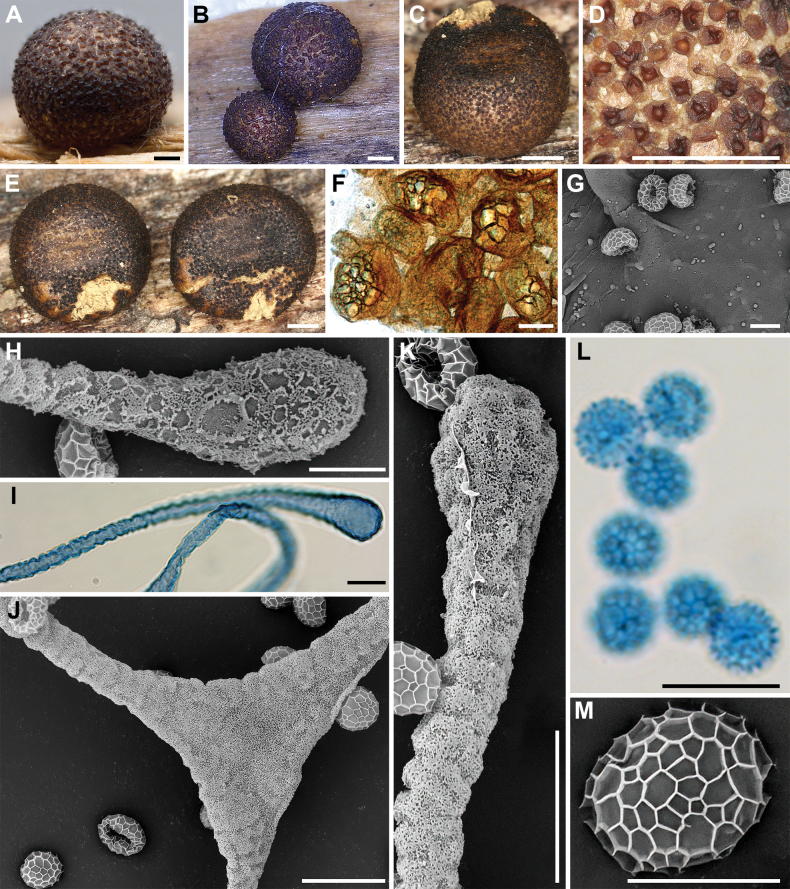
Morphological characteristics of *Lycogalaconvexum*: **A–C** sporocarps; **D** the outer surface of the peridium; **E** sporocarps; **F** peridial vesicles; **G** the inner surface of the peridium (SEM); **H** the detail of capillitium ornamentation (SEM); **I** capillitium; **J** the branch of capillitium (SEM); **K** the detail of capillitium ornamentation (SEM); **L** spores; **M** spore (SEM). Scale bars: 1.5 mm (**A, B**); 1 mm (**C–E**); 100 μm (**F**); 5 μm (**G, H, M**); 10 μm (**I–L**).

#### ﻿ Lycogala epidendrum

Taxon classificationFungiLicealesTubiferaceae

(L.) Fr., Syst. mycol. 3(1):80 (1829) sensu Leontyev et al., Mycol. 115(4):548 (2023)

F2A8BD27-F05D-5E87-9FDF-61FB4DBE326D

MycoBank No: 205910

##### Specimen examined.

A total of 68 specimens. Detailed information on the voucher, geographic location, date of collection, and collectors of studied collections is provided in the Table 1.

##### Distribution.

Widely distributed worldwide.

##### Notes.

*Lycogalaepidendrum* has an extensive distribution and the most variable morphology within the genus (Leontyev et al. 2023b). In this study, out of 163 specimens of *Lycogala* from subtropical China, 68 specimens were identified as *L.epidendrum*, with further molecular confirmation of the identification (Suppl. material 6). The most obvious characteristic of *L.epidendrum* is the presence of very pale, irregular vesicles, faintly visible under RL, as well as the whitish strands of dried slime between the vesicles (Leontyev et al. 2023b). In addition, large sporocarps, rather irregular in shape and forming vast groups, may distinguish the *L.epidendrum*. However, the morphology of *L.epidendrum* exhibits polymorphism. Some of our collections of *L.epidendrum* consist of a few small sporocarps. This means that although the definition of *L.epidendrum* has been narrowed down, it is still a large species complex. This is further confirmed in the species delimitation results of ASAP. Five of ten partitions created by this service separate *L.epidendrum* into several species (Suppl. material 7). More studies, including recombination tests and multigene approaches, are needed to clarify whether *L.epidendrum* is a species complex or complex species.

#### ﻿ Lycogala exiguum

Taxon classificationFungiLicealesTubiferaceae

Morgan, J. Cincinnati Soc. Nat. Hist. 15(3–4):134 (1893)

3C1A02D4-180C-50C5-A3BC-7929BC4C89EF

MycoBank No: 212013

##### Distribution.

Widely distributed worldwide.

##### Specimen examined.

CHINA • Hubei Province: Houhe National Nature Reserve, on rotten wood, 26 Jun 2019, collected by Shu-Zhen Yan and Min Li (HFNNU 11333).

##### Notes.

*Lycogalaexiguum* is considered a species complex at the current level of knowledge (Leontyev et al. 2023b). In the two-gene phylogenetic tree (Fig. 2), collections identified as *L.exiguum*, based on morphological characteristics, formed a large clade represented by at least six putative species. However, it is still uncertain which clade represents the “true” *L.exiguum*, as the DNA data of the type specimen are not available. In this study, we describe two new species (*L.annulatum* and *L.fasciculovesiculiferum*) from the *L.exiguum* complex, both of which differ in morphological characteristics from the original (Morgan 1893) and emended (Leontyev et al. 2023b) descriptions of *L.exiguum*. One more species of the complex, *L. “microcapum*”, will be described in a separate paper.

#### ﻿ Lycogala fasciculovesiculiferum

Taxon classificationFungiLicealesTubiferaceae

W. L. Song, Min Li & Shuang L. Chen sp. nov.

7F4CD511-A0C2-527A-87D6-A37635AC8D53

MycoBank No: 857341

Fig. 7

##### GenBank accession numbers.

PQ685945 (*SSU*).

##### Etymology.

*Fasciculus* (Latin) small bundle or bunch, and *vesicula* (Latin) vesicle, referring to the clustered peridial vesicles.

##### Diagnosis.

Differs from *L.exiguum* by darker peridium, rounded opaque multilayer aggregates of vesicles, and thin vesicle walls with no outer layer covering the whole group.

##### Description.

Sporocarps scattered or in small tight groups, spherical to vertically or horizontally oval, sometimes deformed by mutual pressure, 1.4–2.5 mm in diameter. Peridium membranous, yellowish brown with olivaceous tones or darker, with dense vesicle cover in the upper part of sporocarp, while the lower part is covered much more sparsely. The inner surface of the peridium smooth. Vesicles appear black under RL, deep warm brown or lighter under TL, (31–)33–40(–50) μm in diameter, semi-translucent, forming rounded multilayer aggregates, with 4–7 vesicles across the group and central part opaque due to the overlapping of vesicles. Crystals and oil droplets not observed. Capillitium (2.9–)4.2–5.6(–7.4) μm in diameter, hyaline under TL, with wavy contour, lacking bracelet-like thickening or with very weak ones, densely decorated with small warts and pits. Spore mass, as seen in old collections, yellow with ochraceous undertones or darker, hyaline under TL, (5.4–)5.7–5.9(–6.2) μm in diameter, reticulate, with 5–6 meshes across diameter, unornamented area occupies 1/4 of the spore surface. Plasmodium unknown.

##### Distribution.

Currently known only from China.

##### Habitat.

On rotten wood.

##### Holotype.

CHINA • Henan Province: Baotianman National Nature Reserve, 33.5172°N, 111.9506°E, on rotten wood, 25 Sep 2016, collected by Yang Gao and Gao-Wei Wang (HFNNU 11281).

##### Additional specimens examined.

CHINA • Hubei Province: Houhe National Nature Reserve, 30.0925°N, 110.5932°E, on rotten wood, 20 Jul 2019, collected by Min Li (HFNNU 11274); • Houhe National Nature Reserve, 30.0874°N, 110.6076°E, on rotten wood, 30 Aug 2019, collected by Min Li (HFNNU 11276); • Houhe National Nature Reserve, 30.0879°N, 110.6382°E, on rotten wood, 30 Aug 2019, collected by Min Li (HFNNU 11277); • Houhe National Nature Reserve, 30.0907°N, 110.6210°E, on rotten wood, 30 Aug 2019, collected by Min Li (HFNNU 11278). • Guangdong Province: Dinghu Mountain National Nature Reserve, 23.1798°N, 112.5480°E, on rotten wood, 29 May 2014, collected by Bin Wei and Gang He (HFNNU 11275). • Henan Province: Baotianman National Nature Reserve, 33.5145°N, 111.9492°E, on rotten wood, 25 Sep 2016, collected by Yang Gao and Gao-Wei Wang (HFNNU 11279); • Shunhuang Mountain National Forest Park, 26.4037°N, 111.0553°E, on rotten wood, 13 Jul 2015, collected by Yang Gao and Gao-Wei Wang (HFNNU 11280).

##### Notes.

The small sporocarps, thin capillitium, and densely clustered vesicles may lead to the identification of *L.fasciculovesiculiferum* as *L.exiguum*. However, the vesicle walls in *L.exiguum* are rather thick and multi-layered, with the outer layer covering the whole group, while the vesicle walls in *L.fasciculovesiculiferum* are thin, with no common layer for the group. The shape of vesicle aggregates is mostly elongate in *L.exiguum*, but isodiametric in *L.fasciculovesiculiferum*. Oil droplets in *L.exiguum* are large, one or few in the central part of the vesicle, or small, forming granular deposits. In *L.fasciculovesiculiferum* we did not observe oil accumulations. From a phylogenetic perspective (Fig. 2), the eight specimens representing *L.fasciculovesiculiferum* form a separate clade (UBS = 100, PP = 1). The species status of *L.fasciculovesiculiferum* is supported in all partitions created by ASAP (Suppl. material 7).

**Figure 7.**
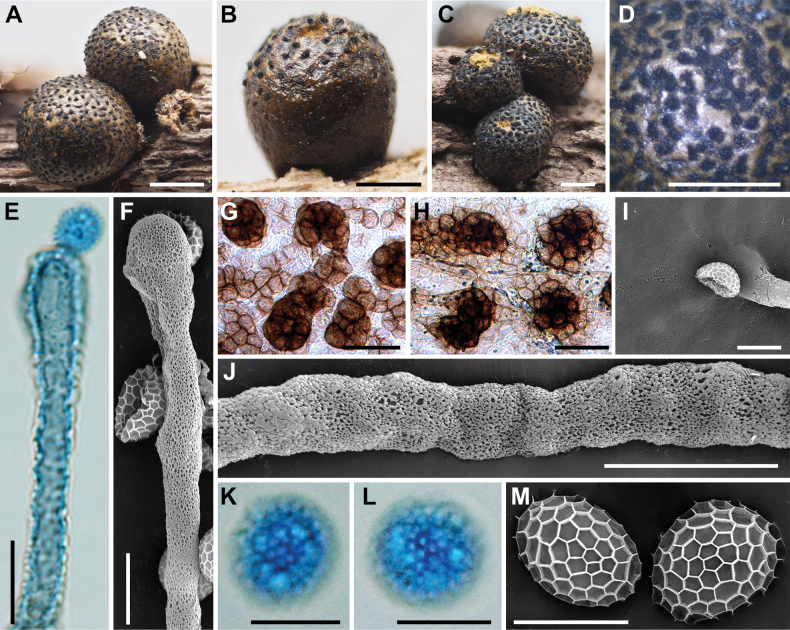
Morphological characteristics of *Lycogalafasciculovesiculiferum*: **A–C** sporocarps; **D** the outer surface of the peridium; **E** capillitium; **F** capillitium (SEM); **G, H** peridial vesicles; **I** the inner surface of the peridium (SEM); **J** the detail of capillitium ornamentation (SEM); **K, L** spore; **M** spores (SEM). Scale bars: 1 mm (**A–D**); 100 μm (**G, H**); 10 μm (**E, J**); 5 μm (**F, I, K–M**).

#### ﻿ Lycogala flavofuscum

Taxon classificationFungiLicealesTubiferaceae

(Ehrenb.) Rostaf., in Fuckel, Jahrb. Nassauischen Vereins Naturk. 27–28:68 (1873)

D06E554B-1B71-50C4-A0A3-D607FECA6F18

MycoBank No: 356737

##### Specimen examined.

NA.

##### Distribution.

Widely distributed worldwide.

##### Notes.

*Lycogalaflavofuscum* has been recorded in Jiangsu Province and Hunan Province in subtropical China (Li et al. 2008). However, no relevant physical specimens were obtained in this study. This species represents a rare case where morphological data alone may be sufficient for reliable identification. Nevertheless, considering the unprecedented hidden diversity of the *Lycogala* species, further confirmation is needed to determine whether subtropical China truly possesses this species.

#### ﻿ Lycogala fossiculatum

Taxon classificationFungiLicealesTubiferaceae

Leontyev, C. Rojas, T. van der Heul, Kochergina & Schnittler, in Leontyev, Ishchenko & Schnittler, Mycologia 115(4):542 (2023)

CEDB4475-F787-5EAB-85BF-326D20FC3D77

MycoBank No: 848155

Fig. 8

##### Specimen examined.

CHINA • Jiangsu Province: Zijin Mountain National Forest Park, 32.0622°N, 118.8761°E, on rotten wood, 18 Jul 2024, collected by Wen-Long Song, Ya-Jing Chen, and Qian Meng (HFNNU 10817). • Jiangxi Province: Guan Mountain National Nature Reserve, 28.5208°N, 114.6127°E, on rotten wood, 13 Sep 2024, collected by Xiao-Dong Liu and Yang Gao (HFNNU 11190); • Guan Mountain National Nature Reserve, 28.5464°N, 114.6315°E, on rotten wood, 8 Aug 2024, collected by Xiao-Dong Liu and Yang Gao (HFNNU 11191).

##### Distribution.

Ukraine, Australia, Costa Rica (Leontyev et al. 2023b), Norway (Johannesen and Vetlesen 2024), and China (this study).

##### Notes.

**Figure 8.**
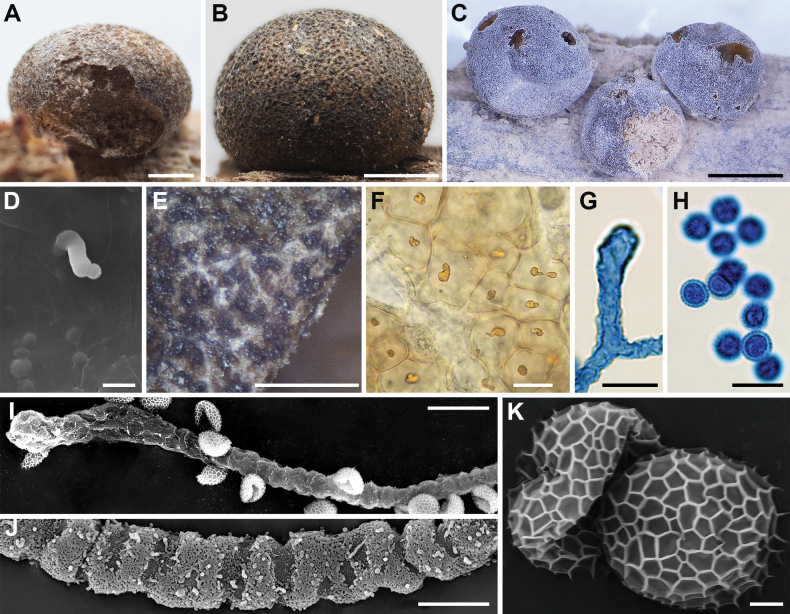
Morphological characteristics of *Lycogalafossiculatum*: **A–C** sporocarps; **D** the inner surface of the peridium (SEM); **E** the outer surface of the peridium; **F** peridial vesicles; **G** the bulbous free ends of the capillitium; **H** spores; **I** the bulbous free ends of the capillitium (SEM); **J** the detail of capillitium ornamentation (SEM); **K** spores (SEM). Scale bars: 1.5 mm (**C**); 1 mm (**A, B, E**); 100 μm (**F**); 10 μm (**G, H, I**); 5 μm (**J**); 2 μm (**D**); 1 μm (**K**).

*Lycogalafossiculatum* is recorded in China for the first time. The main characteristics of this species are large and dark brown sporocarps, accreted peridial vesicles 20–50 μm in diameter, which are rounded, light brownish under TL, filled with spherical oil droplets, and light pinkish-gray or light salmon-gray fresh spore mass (Leontyev et al. 2023b). The specimens observed in this study showed slight differences from the original description: the oil droplets in the vesicles are smaller and pigmented with reddish tints, while the spore ornamentation looks somewhat denser (Fig. 8K). However, two-gene phylogenetic analysis (Fig. 2) showed that Chinese specimens (HFNNU 10817, HFNNU 11190, and HFNNU 11191) cluster together with *L.fossiculatum* (CWP4170, TWDH552.0, USJ7502, USJ7513, and USJ7518) with strong support (UBS = 100, PP = 1). Therefore, combining morphological characteristics and phylogenetic analysis (Fig. 2), we can identify our collections as *L.fossiculatum*.

#### ﻿ Lycogala helvolum

Taxon classificationFungiLicealesTubiferaceae

W. L. Song & Shuang L. Chen sp. nov.

0F17DDF3-3651-56E5-A309-387B6D7D852D

MycoBank No: 857340

Fig. 9

##### GenBank accession numbers.

PQ685930 (*SSU*).

##### Etymology.

*Helvus* (Latin) light yellowish-brown, tawny, pale ochre, referring to the color of the outer surface of the peridium.

##### Diagnosis.

Differs from *L.aggregatum* by yellow with ochraceous undertones or darker peridium, larger peridial vesicles (109–)132–159(–185) μm in diameter, and capillitium decorated with dense reticulum.

##### Description.

Sporocarps scattered, more or less spherical, 1.6–2.3 mm in diameter. Peridium relatively thick and hard, yellow with ochraceous undertones or darker, covered with vesicle aggregates that form small spines as seen from the side. The inner surface of the peridium decorated with unevenly distributed small warts. Vesicles (109–)132–159(–185) μm in diameter, black under RL, deep warm brown or darker under TL, angular from mutual pressure, clustered in rounded, nearly opaque aggregates, with 5–7 vesicles across the group, vesicle walls rather indistinct. Crystals and Oil droplets not observed. Capillitium (3.4–)4.3–5.6(–6.8) μm in diameter, with wavy contour, but no bracelet-like rings, ornamented with dense reticulum of warts and pits, free ends are swallowed. Spore mass in old collections gold or beeswax yellow, hyaline under TL, (4.3–)5.5–6.3(–6.7) μm in diameter, reticulate, with 5–7 meshes across diameter, unornamented area occupies 1/4 of the spore surface. Plasmodium unknown.

##### Distribution.

Currently known only from China.

##### Habitat.

On rotten wood.

##### Holotype.

CHINA • Zhejiang Province: Tianmu Mountain National Nature Reserve, 30.3439°N, 119.4481°E, on rotten wood, 17 Jun 2024, collected by Wen-Long Song and Ya-Jing Chen (HFNNU 10835).

##### Additional specimens examined.

CHINA • Jiangsu Province: Zijin Mountain National Forest Park, 32.0868°N, 118.8596°E, on rotten wood, 20 Jul 2024, collected by Wen-Long Song, Qian Meng, and Ya-Jing Chen (HFNNU 10836).

##### Notes.

The rosette-like aggregation of peridial vesicles, small capillitium diameter, small spore size, and similar density of spore ornamentation place *L.helvolum* close to *L.aggregatum*. These two species are also closely related (Fig. 2). However, the following characteristics allow us to distinguish them. (i) Peridial vesicles. *Lycogalahelvolum* has larger peridial vesicles, (109–)132–159(–185) μm in diameter, with 5–7 vesicles across clusters. In *L.aggregatum* the vesicle diameter is 10–100 μm, their groups contain only 1–3 vesicles across diameter. (ii) Capillitium. The capillitium of *L.helvolum* is ornamented by rounded pits, and possesses bulbous free ends, while the capillitium of *L.aggregatum* may also be covered by large (1–3 μm) rings. Morphological differences between *L.helvolum* and *Lycogalauviforme* are described in Notes for the latter species. From a phylogenetic perspective based on *SSU* and * COI* sequences (Fig. 2), the two specimens representing *L.helvolum* form a sister clade to *L.aggregatum* with strong statistical support (UBS = 100, PP = 1). The interspecific genetic distance between *L.helvolum* and *L.aggregatum*, measured based on *SSU* sequences, is 0.20. The separation of *L.helvolum* is supported in 8 of 10 partitions created by ASAP (Suppl. material 7).

**Figure 9.**
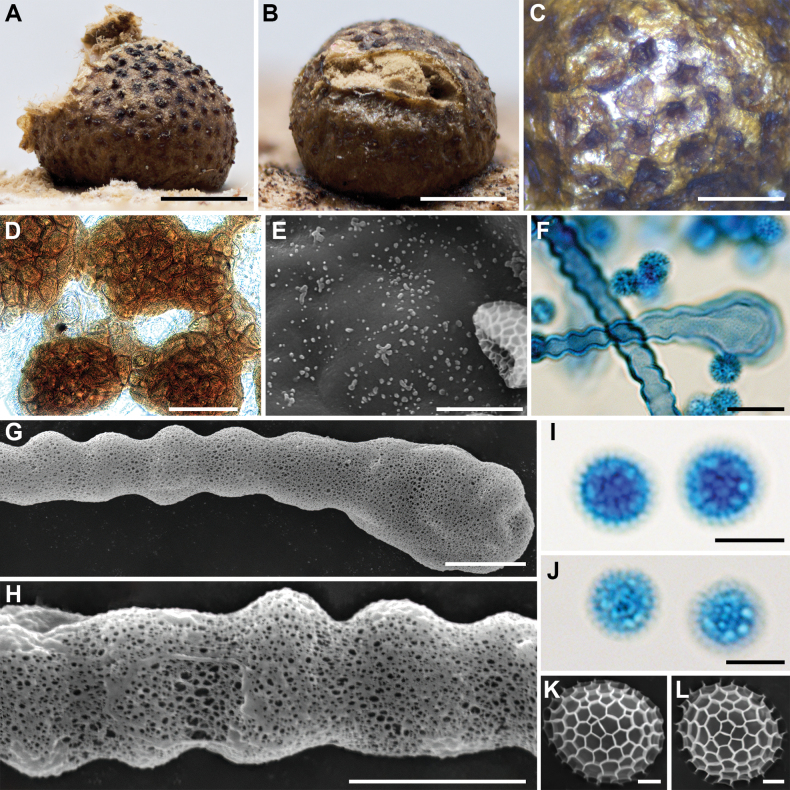
Morphological characteristics of *Lycogalahelvolum*: **A, B** sporocarp; **C** the outer surface of the peridium; **D** peridial vesicles; **E** the inner surface of the peridium (SEM); **F** capillitium; **G** the bulbous free ends of the capillitium (SEM); **H** the detail of capillitium ornamentation (SEM); **I, J** spores (LM); **K, L** spore (SEM). Scale bars: 1 mm (**A–C**); 100 μm (**D**); 10 μm (**F**); 5 μm (**E, G–J**); 1 μm (**K, L**).

#### ﻿ Lycogala indirubinum

Taxon classificationFungiLicealesTubiferaceae

W. L. Song & Shuang L. Chen sp. nov.

0F167726-87B6-594F-8FCE-5DDC35CB713A

MycoBank No: 857342

Fig. 10

##### GenBank accession numbers.

PQ685936 (*SSU*) and PQ728393 (* COI*).

##### Etymology.

*Indigorubin*, based on *indigo* (Greek), blue color from India, and *rubinum* (Latin) ruby-colored, referring to the indigorubin (bluish-purple) tint of the outer surface of the peridium.

##### Diagnosis.

Differs from *L.aggregatum* by almost free thick-walled peridial vesicles and purple tint in pigmentation of sporocarp.

##### Description.

Sporocarps scattered, spherical, short horizontally oval, or irregular, 2.0–5.0 mm in diameter. Peridium thin, membranous, indigorubin (bluish-purple) or lighter, with dense vesicle cover, making it almost black. The inner surface of the peridium is nearly smooth, with only few small warts. Peridial vesicles appear almost black under RL, muted olive-brown or lighter, (78–)106–128(–158) μm in diameter, irregularly ovoid, isodiametric, mostly solitary but densely distributed and closely adjoining. Oil droplets in form of amorphous granular matter or large vesicles, which occupy 1/3–1/2 or vesicle space. Crystals absent. Capillitium (3.7–)5.0–10.0(–21.8) μm in diameter, variable, either completely smooth, or ornamented with large ring-like islets, or densely covered with small pits, thinner tubules are mainly smooth and significantly enlarged to the end, while thicker tubules are mostly covered with pits and their ends are not swollen. Spore mass in old collections yellow with ochraceous undertones or darker, hyaline under TL, (4.8–)5.7–6.3(–7.3) μm in diameter, reticulate, with 4–6 meshes across diameter, unornamented area occupies 1/4 of the spore surface. Plasmodium unknown.

##### Distribution.

Currently known only from China.

##### Habitat.

On rotten wood.

##### Holotype.

CHINA • Hubei Province: Houhe National Nature Reserve, 30.0893°N, 110.5666°E, on rotten wood, 17 Oct 2019, collected by Min Li (HFNNU 11270).

##### Additional specimens examined.

CHINA • Chongqing City: Jinfo Mountain National Nature Reserve, 29.0470°N, 107.1529°E, on rotten wood, 14 Aug 223, collected by Shu-Zhen Yan and Shuang-Lin Chen (HFNNU 11282).

##### Notes.

Fine and nearly smooth capillitium distinguishes *L.indirubinum* from most *Lycogala* species. A similar capillitium also appears in *L.exiguum*, which, however, has densely aggregated peridial vesicles. Nearly black sporocarps, as well as dark solitary vesicles, make *L.indirubinum* similar to *L.maculatum*. However, in the latter species, the capillitium is ornamented with regular bracelet-like thickenings, vesicles do not contain abundant oil, and the peridium lacks the purple indirubin tint. From a phylogenetic perspective (Fig. 2), *L.indirubinum* forms a separate clade (UBS = 100, PP = 1) sister to *L.epidendrum*, *L.maculatum*, *L.projectum*, and *L.irregulare* (UBS = 86, PP = 1). The interspecific genetic distance between *L.indirubinum* and *L.epidendrum*, measured for *SSU* sequences, is 0.20. The separation of *L.indirubinum* is supported in all partitions created by ASAP (Suppl. material 7).

**Figure 10.**
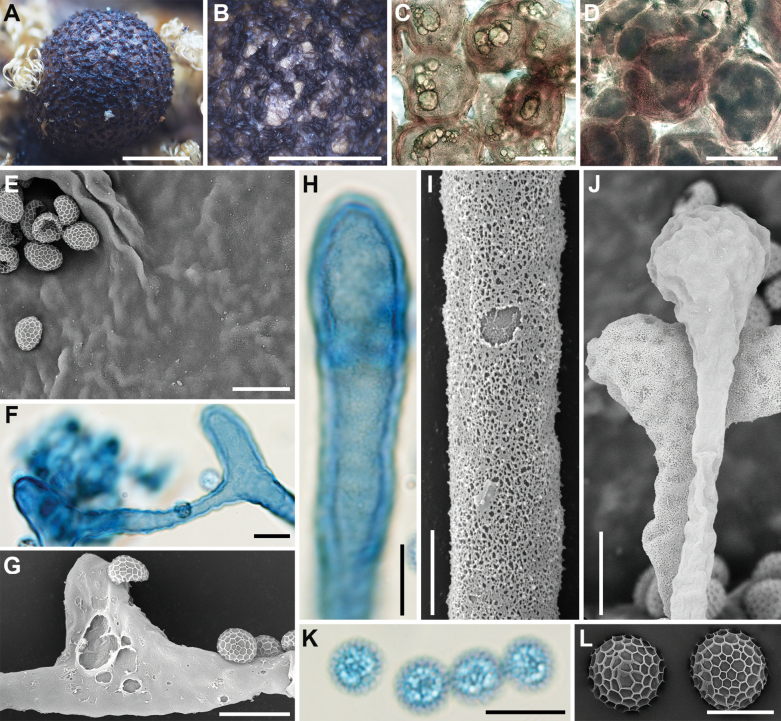
Morphological characteristics of *Lycogalaindirubinum*: **A** sporocarp; **B** the outer surface of the peridium; **C, D** peridial vesicles; **E** the inner surface of the peridium (SEM); **F** capillitium; **G** the detail of capillitium ornamentation (SEM); **H** the bulbous free ends of the capillitium; **I, J** the detail of capillitium ornamentation (SEM); **K** spores; **L** spores (SEM). Scale bars: 1 mm (**A, B**); 100 μm (**C, D**); 10 μm (**E–H, J, K**); 5 μm (**I, L**).

#### ﻿ Lycogala irregulare

Taxon classificationFungiLicealesTubiferaceae

Leontyev, Schnittler, Ishchenko & G. Konstantinides, in Leontyev, Ishchenko & Schnittler, Mycologia 115(4):529 (2023)

6921931C-9681-5EDB-AC93-C590CED7B32D

MycoBank No: 848146

##### Specimen examined.

NA.

##### Distribution.

Ukraine, Russia, Belarus, Germany, Greece (Leontyev et al. 2023b), Norway (Johannesen and Vetlesen 2024), and China (Song et al. 2024b).

##### Notes.

This species was reported from Tiantangzhai National Forest Park, Anhui Province by Song et al. (2024b).

#### ﻿ Lycogala maculatum

Taxon classificationFungiLicealesTubiferaceae

Leontyev, Schnittler & Ishchenko, in Leontyev, Ishchenko & Schnittler, Mycologia 115(4):527 (2023)

9684C822-24CF-5117-BE2D-9AE4D041F8B4

MycoBank No: 848144

##### Specimen examined.

CHINA • Jiangxi Province: Guan Mountain National Nature Reserve, on rotten wood, 12 Sep 2024, collected by Xiao-Dong Liu and Yang Gao (HFNNU 11167); • Guan Mountain National Nature Reserve, on rotten wood, 12 Sep 2024, collected by Xiao-Dong Liu and Yang Gao (HFNNU 11168); • Wuyi Mountain National Park, on rotten wood, 31 Jul 2023, collected by Yang Gao (HFNNU 11174); • Wuyi Mountain National Park, on rotten wood, 31 Jul 2023, collected by Yang Gao (HFNNU 11175); • Guan Mountain National Nature Reserve, on rotten wood, 24 Aug 2020, collected by Xiao-Dong Liu and Yang Gao (HFNNU 11188); • Guan Mountain National Nature Reserve, on rotten wood, 24 Aug 2020, collected by Xiao-Dong Liu and Yang Gao (HFNNU 11189); • Qiyun Mountain National Nature Reserve, on rotten wood, 2 Jul 2024, collected by Yang Gao (HFNNU 11193); • Qiyun Mountain National Nature Reserve, on rotten wood, 2 Jul 2024, collected by Yang Gao (HFNNU 11194); • Lu Mountain National Nature Reserve, 29.5589°N, 115.9760°E, on rotten wood, 16 Jul 2022, collected by Yang Gao (HFNNU 11195); • Lu Mountain National Nature Reserve, 29.5682°N, 116.0030°E, on rotten wood, 16 Jul 2022, collected by Yang Gao (HFNNU 11196); • Guan Mountain National Nature Reserve, 28.5428°N, 114.6326°E, on rotten wood, 7 Aug 2024, collected by Xiao-Dong Liu (HFNNU 11222). • Guizhou Province: Changpoling National Nature Reserve, 26.6622°N, 106.6771°E, on rotten wood, 20 Aug 2023, collected by Yang Gao (HFNNU 11250); • Changpoling National Nature Reserve, 26.6658°N, 106.6879°E, on rotten wood, 20 Aug 2023, collected by Yang Gao (HFNNU 11251).

##### Distribution.

Ukraine, Russia, Vietnam, Germany (Leontyev et al. 2023b), Belarus (Leontyev et al. 2023b; Leontyev et al. 2024), Norway (Johannesen and Vetlesen 2024), and China (Leontyev et al. 2023b, and this study).

##### Notes.

*Lycogalamaculatum* was separated from *L.epidendrum* complex based on morphological characteristics and two-gene phylogenetic analysis (*SSU* and * COI*) by Leontyev et al. (2023a, b). The first record of *L.maculatum* in China was in Shunhuang Mountain National Forest Park, Hunan Province (Leontyev et al. 2023b). In this study, we provide new data on the distribution of this species in China, including Jiangxi Province and Guizhou Province (Table 1). Peridium in this species is covered with large, solitary, dark brown vesicles and is often dark itself (Leontyev et al. 2023b). Compared to similar species, *L.alisaulianovae* and *Lycogalaroseosporum*, the color of the spore mass of *L.maculatum* is gray, later warm gray, in old specimens yellowish-gray (Leontyev et al. 2023b), while the color of the spore mass of *L.alisaulianovae* and *L.roseosporum* is bright pink and bluish, respectively. In our material, the color of the spore mass of *L.maculatum* sometimes appeared rather bright pink, although not as saturated as in *L.roseosporum*. Therefore, the range of variation of the color of the spore mass in *L.maculatum* needs further exploration.

#### ﻿ Lycogala nigrum

Taxon classificationFungiLicealesTubiferaceae

W. L. Song & Shuang L. Chen sp. nov.

282F5BB1-BAE2-5BE2-86F8-3896751305C5

MycoBank No: 857339

Fig. 11

##### GenBank accession numbers.

PQ685932 (*SSU*).

##### Etymology.

*Niger* (Latin) black, referring to very dark, opaque peridial vesicles.

##### Diagnosis.

Differs from all other species in the genus by possessing peridial vesicles that appear black and opaque under transmitted light.

##### Description.

Sporocarps scattered, spherical, short horizontally oval, sometimes deformed by mutual pressure, 1.7–3.7 mm in diameter. Peridium membranous, thin, yellowish brown with olivaceous tones or lighter under RL, with dense vesicle cover, giving a nearly black appearance, the dehiscence area at the top of sporocarp conspicuous, free of vesicles, irregular in shape. The inner peridial surface smooth or covered with scattered warts. Vesicles black under RL, dark under TL, (117.6–)161.7–237.8(–269.2) μm in diameter, isodiametric, irregularly polygonal, solitary, but densely distributed. Oil droplets present, large or small, irregular in shape, reddish, concentrated in the central part of vesicles. Capillitium tubular, hyaline under TL, (5.1–)5.3–9.1(–19.8) μm in diameter, with smooth or somewhat wavy contour and no bracelet-like thickening, the surface of the thread is decorated with small warts and pits, and occasional ling-like islets, visible under TL and SEM. Spore mass in old collections yellow with ochraceous undertones or darker, hyaline under TL, (5.7–)6.0–6.2(–6.4) μm in diameter, reticulate, with 6–7 meshes across diameter, unornamented area occupies 1/4 of the spore surface. Plasmodium unknown.

##### Distribution.

Currently known only from China.

##### Habitat.

On rotten wood.

##### Holotype.

CHINA • Jiangsu Province: Zijin Mountain National Forest Park, 32.0628°N, 118.8761°E, on rotten wood, 10 Jun 2015, collected by Gao-Wei Wang and Shu-Zhen Yan (HFNNU 11266).

##### Additional specimens examined.

CHINA • Anhui Province: Huang Mountain National Forest Park, 30.1421°N, 118.2071°E, on rotten wood, 5 Jul 2008, collected by Shuang-Lin Chen (HFNNU 11267).

##### Notes.

To our knowledge, among all 23 described species of *Lycogala* (Lado 2005–2025), there is not a single species in which vesicles appear black and opaque under TL (Leontyev et al. 2023b). This makes *L.nigrum* unique in this regard. From a phylogenetic perspective (Fig. 2), the two specimens representing *L.nigrum* form a separate clade (UBS = 100, PP = 1) related to *L.alisaulianovae* and *L.roseoporum* (UBS = 76). These two species share dark solitary vesicles with *L.nigrum*, but in both of them these structures are not opaque. The interspecific genetic distance between *L.nigrum* and the other species in this clade, measured based on *SSU* sequences, is 0.36. The separation of *L.nigrum* is supported in all partitions created by ASAP (Suppl. material 7).

**Figure 11.**
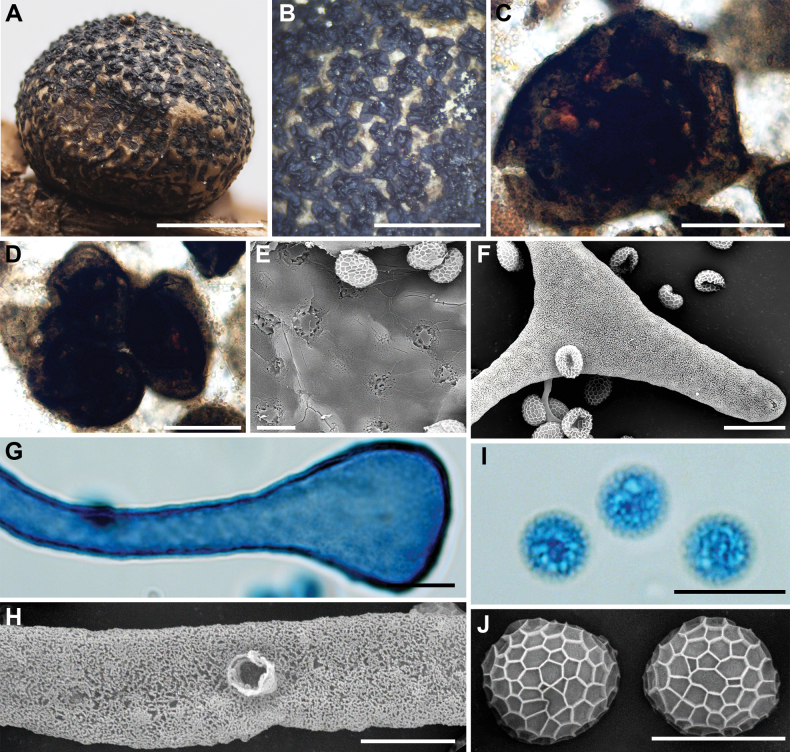
Morphological characteristics of *Lycogalanigrum*: **A** sporocarp; **B** the outer surface of the peridium; **C, D** peridial vesicles; **E** the inner surface of the peridium (SEM); **F** the branch of the capillitium (SEM); **G** capillitium; **H** the detail of capillitium ornamentation (SEM); **I** spores; **J** spores (SEM). Scale bars: 1.5 mm (**A**); 1 mm (**B**); 100 μm (**C, D**); 5 μm (**E, H, J**); 10 μm (**F, G, I**).

#### ﻿ Lycogala planovesiculiferum

Taxon classificationFungiLicealesTubiferaceae

W. L. Song, Z. Q. Jiang & Shuang L. Chen sp. nov.

7F6435AB-7287-526A-B560-C45424A3CCC6

MycoBank No: 857336

Fig. 12

##### GenBank accession numbers.

PQ685819 (*SSU*) and PQ728314 (* COI*).

##### Etymology.

*Planus* (Latin) flat, and *vesicula* (Latin) vesicle, referring to the flattened vesicles on the outer surface of the peridium.

##### Diagnosis.

Differs from *L.alisaulianovae* and *L.roseosporum* by light deep warm brown spore mass and small, flat peridial vesicles.

##### Description.

Sporocarps scattered, spherical, short horizontally oval, or somewhat irregular, 1.7–2.9 mm in diameter. Peridium thin, membranous, muted olive-brown or lighter, evenly covered with flattened vesicles. Inner peridial surface smooth or covered with scattered small warts. Vesicles brown under RL, light ochre or lighter, circular, or shuttle-shaped under TL, or 2–3 connected, (51–)63–77(–104) μm in diameter, containing irregular oil droplets. Capillitium tubular, (4.8–)5.7–7.3(–13.8) μm in diameter, hyaline under TL, with wavy contour and weak bracelet-like thickenings, free ends more or less swollen. The surface of capillitium ornamented with rather chaotic reticulum of warts and pits, and occasional ring-like islets. Spore mass in old collections rich warm red or lighter, hyaline under TL, (5.9–)6.5–6.8(–7.1) μm in diameter, reticulate, with 4–5 meshes across diameter, unornamented area occupies 1/4 of the spore surface. Plasmodium unknown.

##### Distribution.

Currently known only from China and Vietnam (Leontyev et al. 2023a).

##### Habitat.

On rotten wood.

##### Holotype.

CHINA • Zhejiang Province: Tianmu Mountain National Nature Reserve, 30.3233°N, 119.4562°E, on rotten wood, 17 Jun 2024, collected by Wen-Long Song and Ya-Jing Chen (HFNNU 10819).

##### Additional specimens examined.

CHINA • Zhejiang Province: Tianmu Mountain National Nature Reserve, 30.3496°N, 119.4466°E, on rotten wood, 18 Jun 2024, collected by Wen-Long Song and Ya-Jing Chen (HFNNU 10820); • Tianmu Mountain National Nature Reserve, 30.3469°N, 119.4411°E, on rotten wood, 18 Jun 2024, collected by Wen-Long Song and Ya-Jing Chen (HFNNU 10821). • Anhui Province: Huang Mountain National Forest Park, 30.1573°N, 118.1930°E, on rotten wood, 5 Jul 2008, collected by Shuang-Lin Chen (HFNNU 11346).

##### Notes.

Small sporocarps covered by solitary drop-like vesicles, as well as capillitium decorated with small pits and warts, make *L.planovesiculiferum* similar to *L.alisaulianovae* and *L.roseosporum*. However, these two species have different pigmentation of spore mass, bluish-gray and bright pink, respectively. Vesicles in *L.planovesiculiferum* are smaller than in both related species (50–105 µm vs. 100–300 µm in *L.alisaulianovae* and *L.roseosporum*). From a phylogenetic perspective (Fig. 2), the specimens representing *L.planovesiculiferum* form a sister group with *L.oncoides* but with low statistical support. These two species do not seem closely related, since *L.oncoides* has aggregated oil-containing peridial vesicles. The separation of *L.planovesiculiferum* is supported in all partitions created by ASAP (Suppl. material 7).

**Figure 12.**
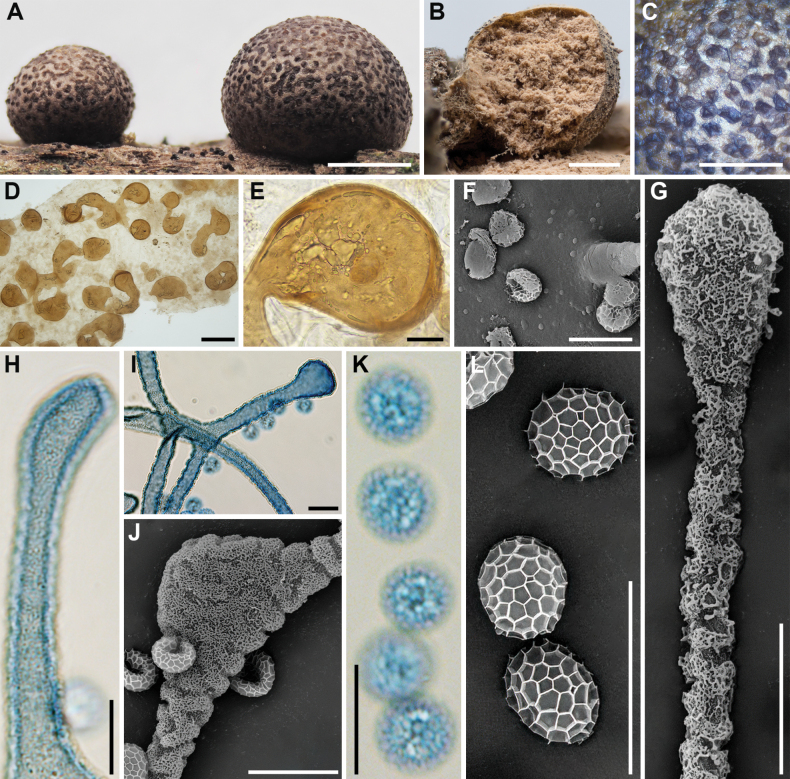
Morphological characteristics of *Lycogalaplanovesiculiferum*: **A** sporocarps; **B** spore mass; **C** the outer surface of the peridium; **D, E** peridial vesicles; **F** the inner surface of the peridium (SEM); **G** the bulbous free ends of the capillitium (SEM); **H** the bulbous free ends of the capillitium; **I** capillitium; **J** the detail of capillitium ornamentation (SEM); **K** spores; **L** spores (SEM). Scale bars: 1 mm (**A–C**); 100 μm (**D**); 20 μm (**E**); 10 μm (**F–L**).

#### ﻿ Lycogala projectum

Taxon classificationFungiLicealesTubiferaceae

W. L. Song, Yang Gao & Shuang L. Chen sp. nov.

D33F7257-BCED-58E8-B769-7597E33FD8D8

MycoBank No: 857343

Fig. 13

##### GenBank accession numbers.

PQ685914 (*SSU*) and PQ728376 (* COI*).

##### Etymology.

*Projectum* (Latin) projected, jutting out, referring to the vesicles protrude outward from outer surface of the peridium, and forming distinct large spines.

##### Diagnosis.

Differs *L.maculatum* by smaller peridial vesicles, thinner capillitium, and smaller spores.

##### Description.

Sporocarps scattered, spherical, short horizontally oval, or somewhat irregular, 2.3–5.3 mm in diameter. Peridium thick, relatively hard, dried rose or darker, covered by vesicles, which are denser in the upper part. The inner surface of the peridium ornamented with warts, spins, and innumerous parallel lines. Vesicles protrude outward from the surface of the peridium, forming distinct large spines, black under RL, deep warm brown or lighter under TL, solitary, free or occasionally connecting to each other, irregular rounded, (49–)66–117(–140) μm in diameter. Crystals and oil droplets absent. Capillitium tubular, near hyaline under TL, (2.6–)3.0–4.3(–5.4) μm in diameter, with prominent, regularly situated, smooth or warty bracelet-like thickening, less obvious near connection between the capillitium and the peridium or at the free end. Free ends of tubules bulbous, reaching 7.4 μm in diameter. Spore mass in old collections yellow with ochraceous undertones or darker, hyaline under TL, (5.0–)5.8–6.4(–6.7) μm in diameter, reticulate, with 4–6 meshes across diameter, unornamented area occupies 1/4–1/2 of the spore surface. Plasmodium unknown.

##### Distribution.

Currently known only from China.

##### Habitat.

On rotten wood.

##### Holotype.

CHINA • Anhui Province: Tiantangzhai National Forest Park, on rotten wood, 31.1584°N, 115.7803°E, 26 Jul 2016, collected by Gao-Wei Wang and Yang Gao (HFNNU 11248).

##### Additional specimens examined.

CHINA • Jiangxi Province: Jinggang Mountain National Nature Reserve, 26.6727°N, 114.0616°E, on rotten wood, 8 Jul 2020, collected by Yang Gao (HFNNU 11247). • Anhui Province: Tiantangzhai National Forest Park, 31.1532°N, 115.7838°E, on rotten wood, 26 Jul 2016, collected by Gao-Wei Wang and Yang Gao (HFNNU 11249).

##### Notes.

This species is similar to and closely related to *L.maculatum* (Leontyev et al. 2023b). Both taxa share solitary dark-brown vesicles and prominent bracelet-like thickening on the capillitium tubules. However, the peridial vesicles in *L.projectum* are somewhat smaller (50–140 μm vs. 100–230 μm), capillitium is thinner (2–5 μm vs. 5–20 μm), spores are smaller ((5.0–)5.8–6.4(–6.7) μm vs. (6–)6.5–8.5(–9.5) μm). In addition, the ornamentation of the inner surface of the peridium in *L.projectum* seems to be unique: it is densely covered by warts, spines, and parallel lines. From a phylogenetic perspective (Fig. 2), *L.projectum* and *L.maculatum* form a monophyletic clade with strong statistical support (UBS = 100, PP = 1). The interspecific genetic distance between these two species, measured for *SSU* sequences, is 0.15. The separation of *L.projectum* is supported in 8 of 10 partitions created by ASAP (Suppl. material 7).

**Figure 13.**
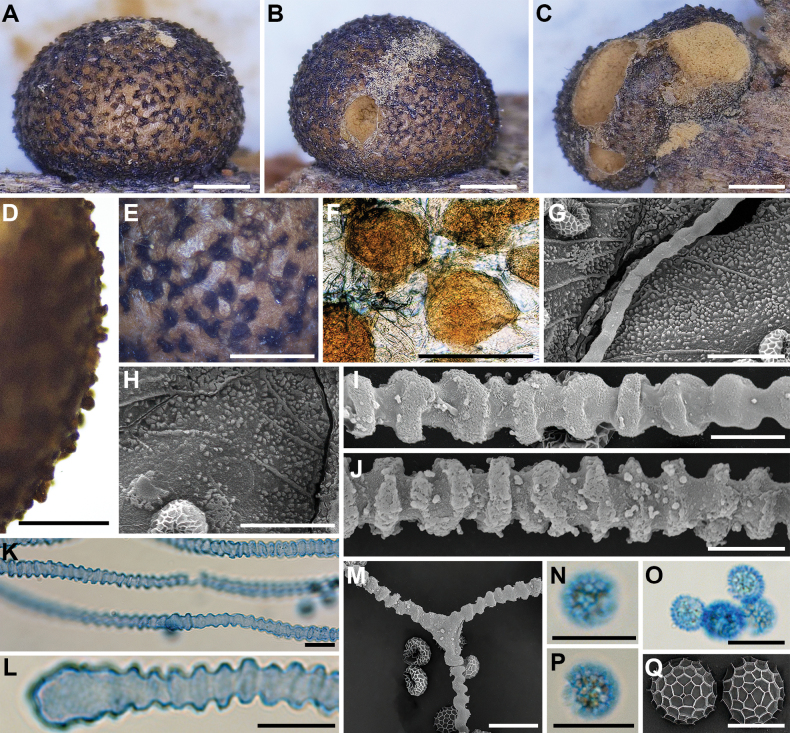
Morphological characteristics of *Lycogalaprojectum*: **A–C** sporocarp; **D** the edge of sporocarp highlights the large spines formed by the peridial vesicles; **E** the outer surface of the peridium; **F** peridial vesicles; **G, H** the inner surface of the peridium (SEM); **I, J** the detail of capillitium ornamentation (SEM); **K** capillitium; **L** the bulbous free ends of the capillitium; **M** the branch of the capillitium (SEM); **N–P** spores; **Q** spores (SEM). Scale bars: 1 mm (**A–E**); 100 μm (**F**); 10 μm (**G, H, K–P**); 5 μm (**I, J, Q**).

#### ﻿ Lycogala skovorodaense

Taxon classificationFungiLicealesTubiferaceae

Leontyev, Schnittler & Ishchenko, in Leontyev, Ishchenko & Schnittler, Mycologia 115(4):542 (2023)

2E33B453-59B2-5733-818E-0DDE538E0DC5

MycoBank No: 848154

Fig. 14

##### Specimen examined.

CHINA • Zhejiang Province: Tianmu Mountain National Reserve, 30.3299°N, 119.4543°E, on rotten wood, 18 Jun 2024, collected by Wen-Long Song and Ya-Jing Chen (HFNNU 9993). • Jiangsu Province: Zijin Mountain National Forest Park, 32.0806°N, 118.8444°E, on rotten wood, 20 Jul 2024, collected by Wen-Long Song, Qian Meng, and Ya-Jing Chen (HFNNU 10815); • Zijin Mountain National Forest Park, 32.0769°N, 118.8447°E, on rotten wood, 20 Jul 2024, collected by Wen-Long Song, Qian Meng, and Ya-Jing Chen (HFNNU 10816); • Zijin Mountain National Forest Park, 32.0831°N, 118.8402°E, on rotten wood, 20 Jul 2024, collected by Wen-Long Song, Qian Meng, and Ya-Jing Chen (HFNNU 11225). • Hubei Province: Houhe National Nature Reserve, 30.0808°N, 110.5808°E, on rotten wood, 30 Aug 2019, collected by Min Li (HFNNU 11220).

##### Distribution.

Ukraine, Russia, Germany (Leontyev et al. 2023b), and China (this study).

##### Notes.

**Figure 14.**
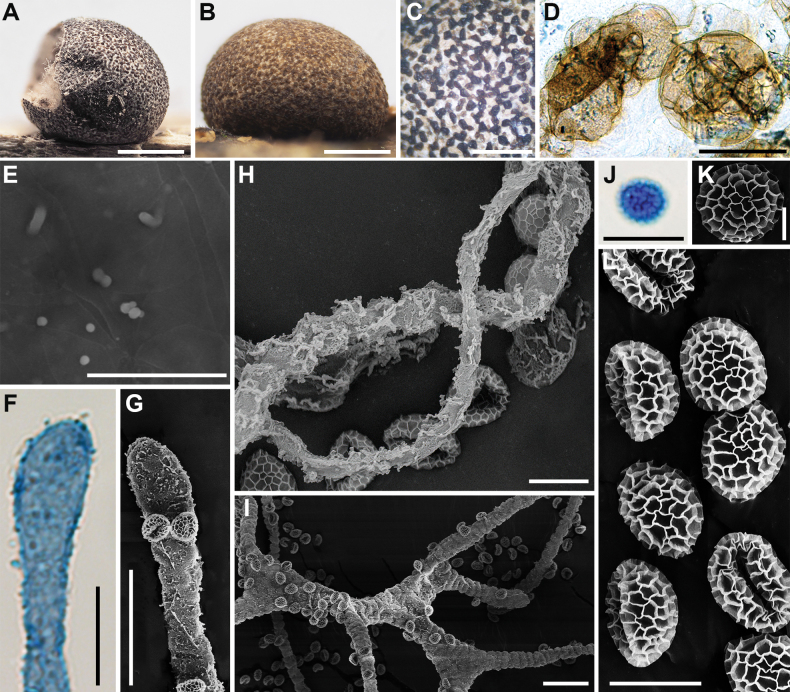
Morphological characteristics of *Lycogalaskovorodaense*: **A, B** sporocarp; **C** the outer surface of the peridium; **D** peridial vesicles; **E** the inner surface of the peridium (SEM); **F** the bulbous free ends of the capillitium; **G** the bulbous free ends of the capillitium (SEM); **H** the detail of capillitium ornamentation (SEM); **I** the branch of the capillitium (SEM); **J** spore; **K, L** spores (SEM). Scale bars: 1 mm (**A, C**); 1.5 mm (**B**); 100 μm (**D**); 20 μm (**G, I**); 10 μm (**E, F, J**); 5 μm (**H, L**); 2 μm (**K**).

*Lycogalaskovorodaense* is recorded in China for the first time. The main characteristics of this species are small, dark brown sporocarps, accreted peridial vesicles, forming dense, rounded, rosette-like clusters, as well as oil accumulations in vesicles, the capillitium ornamented by large warts and spines, the spores mass that is gray or warm gray, and the spores with 4–6 meshes across their diameter (Leontyev et al. 2023b). The Chinese collections show slight differences in capillitium ornamentation compared to the original description (Leontyev et al. 2023b). We observed two types of ornamentation (Fig. 14G–I): one containing ridges and clustered warts (Fig. 14H), and another ornamented with small warts, pits, and irregular ring-like inlets; the latter type is not directly mentioned in the original description. The phylogenetic analysis (Fig. 2) has clustered the Chinese specimens (HFNNU 9993, HFNNU 10815, HFNNU 10816, HFNNU 11220, and HFNNU 11255) with *L.skovorodaense* (IY15, CWP4234, CWP4169, and sc27535) with strong support (UBS = 100, PP = 1).

#### ﻿ Lycogala succineum

Taxon classificationFungiLicealesTubiferaceae

Leontyev & Schnittler, in Leontyev, Ishchenko & Schnittler, Mycologia 115(4):538 (2023)

E0C3AB7C-95C6-5E9D-A049-07F222E982A4

MycoBank No: 848152

Fig. 15

##### Specimen examined.

CHINA • Jiangsu Province: Zijin Mountain National Forest Park, 32.0587°N, 118.8678°E, on rotten wood, 20 Jul 2024, collected by Wen-Long Song, Qian Meng, and Ling-Xiao Chen (HFNNU 10822); • Zijin Mountain National Forest Park, 32.0623°N, 118.8748°E, on rotten wood, 18 Jul 2024, collected by Wen-Long Song, Ya-Jing Chen, and Qian Meng (HFNNU 10828); • Zijin Mountain National Forest Park, 32.0594°N, 118.8657°E, on rotten wood, 20 Jul 2024, collected by Wen-Long Song, Qian Meng, and Ling-Xiao Chen (HFNNU 10833); • Zijin Mountain National Forest Park, 32.0572°N, 118.8693°E, on rotten wood, 20 Jul 2024, collected by Wen-Long Song, Qian Meng, and Ling-Xiao Chen (HFNNU 10834). • Henan Province: Baotianman National Nature Reserve, 33.5129°N, 111.9432°E, on rotten wood, 23 Jul 2016, collected by Yang Gao and Gao-Wei Wang (HFNNU 11166); • Baotianman National Nature Reserve, 33.5152°N, 111.9399°E, on rotten wood, 23 Jul 2016, collected by Yang Gao and Gao-Wei Wang (HFNNU 11200).

##### Distribution.

Ukraine, Germany (Leontyev et al. 2023b), Belarus (Leontyev et al. 2023b; Leontyev et al. 2024), Norway (Leontyev et al. 2023b; Johannesen and Vetlesen 2024), and China (this study).

##### Notes.

*Lycogalasuccineum* is recorded in China for the first time. The main characteristics of this species are medium-sized, rufous beige sporocarps, the vesicles, which are loosely and evenly distributed, solitary, rounded, amber-yellow peridial, and the spore mass, which is light pinkish-gray or light salmon-gray. The Chinese collections fit well with the original description of *L.succineum* (Leontyev et al. 2023b). Subtle differences include the ornamentation of capillitium, which does not possess regular bracelet-like thickenings, as stated in the original description, and the presence of small spines on the surface. However, these differences do not appear significant (see fig. 10H, K in Leontyev et al. 2023b). From the perspective of phylogenetic analysis (Fig. 2), Chinese specimens of *L.succineum* (HFNNU 10822, HFNNU 10828, HFNNU 10833, HFNNU 10834, HFNNU 11166, and HFNNU 11200) cluster together with other specimens of *L.succineum* (UBS = 100, PP = 1).

**Figure 15.**
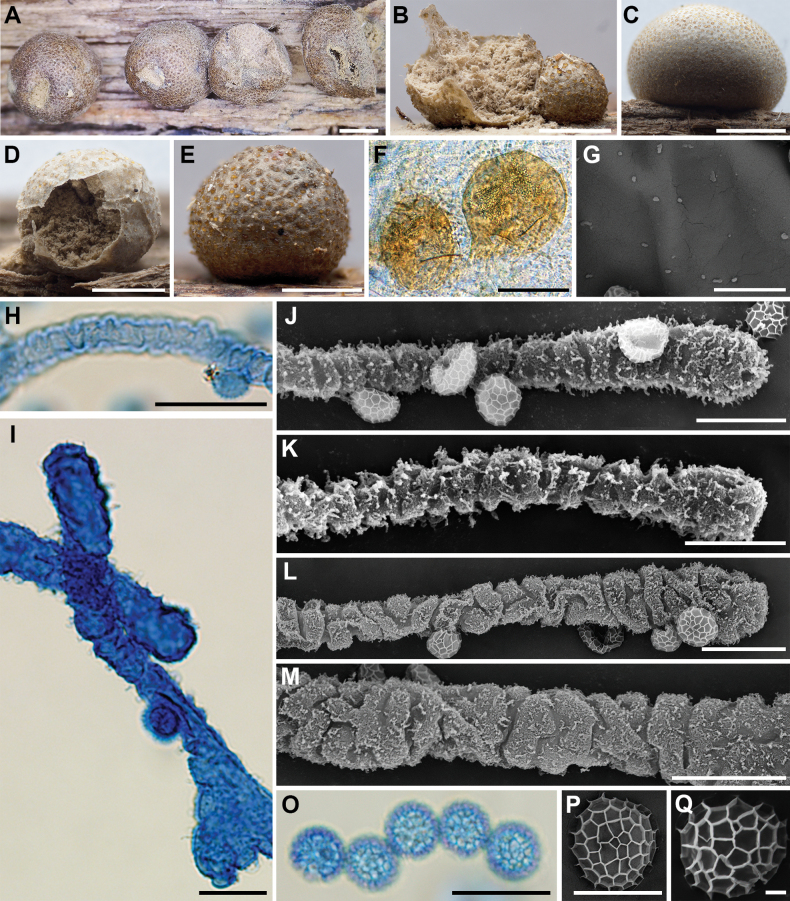
Morphological characteristics of *Lycogalasuccineum*: **A–E** sporocarps; **F** peridial vesicles; **G** the inner surface of the peridium (SEM); **H, I** capillitium; **J–L** the bulbous free ends of the capillitium (SEM); **M** the detail of capillitium ornamentation (SEM); **O** spores; **P**, **Q** spore (SEM). Scale bars: 1 mm (**A–E**); 50 μm (**F**); 20 μm (**H**); 10 μm (**I–O**); 5 μm (**G, P**); 1 μm (**Q**).

#### ﻿ Lycogala uviforme

Taxon classificationFungiLicealesTubiferaceae

W. L. Song, Yang Gao & Shuang L. Chen, Leontyev & Z. Edwards sp. nov.

36C011C6-FC3E-5929-97D7-58752B100022

MycoBank No: 857337

Fig. 16

##### GenBank accession numbers.

PQ685905 (*SSU*).

##### Etymology.

*Uva* (Latin) bunch of grapes, referring to the aggregation of peridial vesicles.

##### Diagnosis.

Differs from *L.botrydium* by sporocarps more or less regular in shape, covered with dark brown reticulum of aggregated vesicles, by thick and distinguishable vesicle walls, and by capillitium densely ornamented by pits, forming a prominent reticulate pattern.

##### Description.

Sporocarps scattered, spherical or slightly ovoid, 1.4–2.9 mm in diameter. Peridium relatively thick, deep warm brown, covered by vesicles that form a dense dark reticulum, through the gaps of which the lighter peridium is visible. The inner surface of the peridium is smooth. Vesicles under RL matt, dark grayish-brown, under TL deep warm brown, ovoid, angular by mutual pressure, (55–)60–85(–100) μm in diameter, clustered in elongate, ovoid to fusiform aggregates with 2–5 vesicles across the group, often merged to form a reticulum. Vesicle walls conspicuous, rather thick (2–5 µm). Crystals absent. Oil droplets numerous, irregular in shape, filling up to half of the vesicles space. Capillitium tubular, hyaline under TL, (3.7–)4.5–6.3(–9.5) μm in diameter, with smooth contours, densely ornamented by numerous pits, which form a prominent reticulate pattern, better visible under SEM. Spore mass in old collections light yellow with ochraceous undertones, hyaline under TL, (5.5–)5.7–6.0(–6.3) μm in diameter, reticulate, with 5–7 meshes across diameter, unornamented area occupies 1/4–1/2 of the spore surface. Plasmodium unknown.

##### Distribution.

Currently known only from China and USA.

##### Habitat.

On rotten wood.

##### Holotype.

CHINA • Hubei Province: Xingshan County, on rotten wood, 24 Jun 2017, collected by Yang Gao (HFNNU 11239).

##### Additional specimens examined.

CHINA • Jiangsu Province: Lao Mountain National Forest Park, 32.1191°N, 118.6341°E, on rotten wood, 24 Jul 2015, collected by Yang Gao (HFNNU 11240). • Hubei Province: Xingshan County, on rotten wood, 24 Jun 2017, collected by Yang Gao (HFNNU 11241). • Jiangxi Province: Lu Mountain National Nature Reserve, 29.5669°N, 115.9941°E, on rotten wood, 16 Jul 2022, collected by Yang Gao (HFNNU 11242). • USA. Maine: Cumberland County, environs of Standish, in conifer area within mixed hardwood, 43.6906°N, 70.6056°W, on dead, barkless hardwood log, 24 Jul 2023, collected by Zachariah Edwards (ZachEdw1).

##### Notes.

*Lycogalauviforme* is similar to and phylogenetically related to *L.botrydium* (Leontyev et al. 2023b), sharing dense ornamentation of the peridium by vesicle aggregates, their fusiform shape, brown pigmentation, and abundant oil content. However, the peridial vesicles of *L.botrydium* have thin, nearly invisible vesicle walls, while in *L.uviforme* they are thick and conspicuous. Sporocarps of *L.botrydium* are irregular in shape and somewhat flattened, while in *L.uviforme*, they are spherical or slightly ovoid. The vesicle ornamentation is clearly reticulate in *L.uviforme* and rather spotted in *L.botrydium*. In addition, the capillitium of *L.botrydium* is fine-pitted and warty, with ring-shaped islets, while in *L.uviforme* it is covered with larger pits, giving a reticulate appearance to its surface. From the phylogenetic perspective (Fig. 2), *L.uviforme* and *L.botrydium* form a monophyletic group that received strong support (UBS = 100, PP = 1). The interspecific genetic distance between these species, measured for *SSU* sequences, is 0.17. The separation of *L.uviforme* is supported in 8 of 10 partitions created by ASAP (Suppl. material 7).

**Figure 16.**
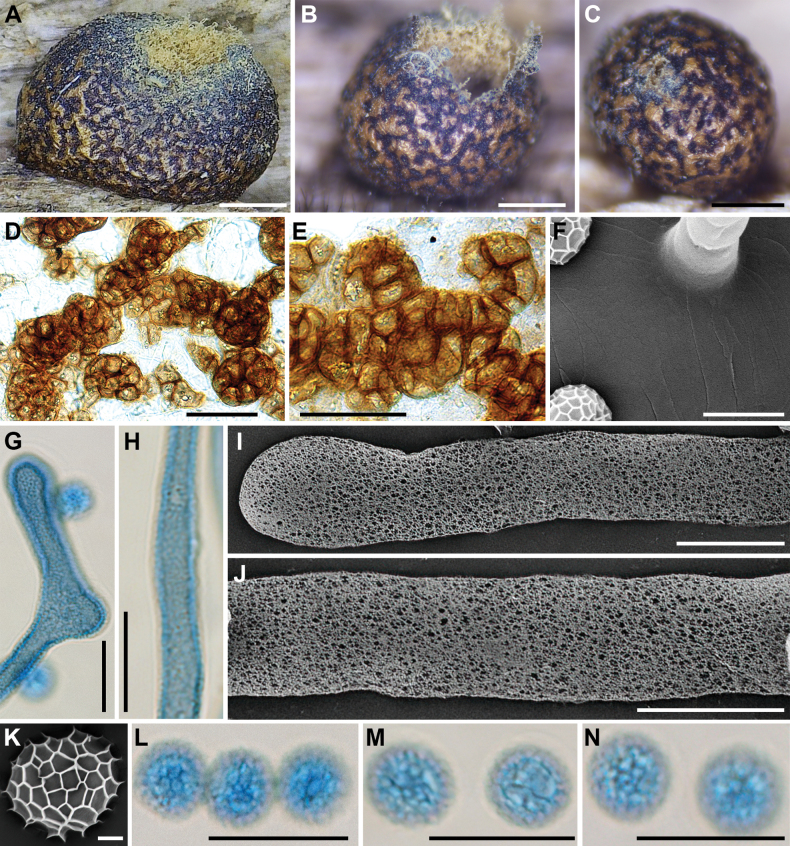
Morphological characteristics of *Lycogalauviforme*: **A–C** sporocarp; **D, E** peridial vesicles; **F** the inner surface of the peridium (SEM); **G** the bulbous free ends of the capillitium; **H** capillitium; **I** the bulbous free ends of the capillitium (SEM); **J** the detail of capillitium ornamentation (SEM); **K** spore (SEM); **L–N** spores. Scale bars: 0.5 mm (**A–C**); 100 μm (**D, E**); 10 μm (**G, H, L–N**); 5 μm (**F, I, L**); 1 μm (**K**).

## ﻿Discussion

### ﻿Diversification of morphological characteristics in *Lycogala*

The description of ten new species not only expands the genus *Lycogala* with new taxa, but also broadens the spectrum of morphological traits known for the genus. For instance, the ornamentation on capillitium with pits, forming a regular net-like ornament (*L.uviforme* and *L.helvolum*), was not previously reported in *Lycogala* (Leontyev and Schnittler 2023). Similarly, opaque peridial vesicles (*L.nigrum*) and ornamentation of the peridium by straight lines (*L.projectum*) introduce novel diagnostic traits in the taxonomy of the genus. These discoveries underscore the importance of meticulous morphological reinvestigation, even in taxa considered relatively well-studied, as subtle yet taxonomically significant features may remain undetected without high-resolution microscopic analysis.

### ﻿Undescribed species

In addition to the species listed above, we recorded several specimens that may represent three species that are not formally described. These are *Lycogala “melleum*” (represented by HFNNU 11284, HFNNU 11285, HFNNU 11287, HFNNU 11288, HFNNU 11289, HFNNU 11283, and HFNNU 11286), *Lycogala “guttatum*” (represented by HFNNU 11269, HFNNU 11268, HFNNU 10830, and HFNNU 10831), and *Lycogala “densum*” (represented by HFNNU 11261, HFNNU 11271, HFNNU 11259, HFNNU 11257, HFNNU 11258, HFNNU 11260, HFNNU 11262, HFNNU 10818, and HFNNU 11256). However, these putative species have not been described in this publication due to a lack of stable morphological differences from known species. As more collections become available, reliable morphological characteristics may be identified, potentially leading to the formal description of these species.

### ﻿Notable species diversity of *Lycogala* in China

In this study, 21 *Lycogala* species from subtropical China were documented, representing a significant contribution to the understanding of the global diversity of this genus. These findings are based on data from only 11 provincial-level administrative regions or municipalities with notably uneven sampling efforts across these areas (Fig. 1, Table 1). Given that China comprises 34 provincial-level administrative divisions, including municipalities, autonomous regions, and special administrative regions, our current dataset likely represents only a small fraction of the actual diversity of *Lycogala* present in the country.

The fact that a relatively small number of collected specimens (163) yielded a considerable number of species, nearly half of which are new to science, suggests that China harbors a substantially richer *Lycogala* species composition than currently documented. These results highlight the need for more extensive and systematic sampling across China’s diverse biogeographical regions. Future research should prioritize under-sampled regions, particularly in western China and areas with specific microclimates, like highlands and deserts, to achieve a more comprehensive understanding of the myxomycete biodiversity within the country. Such efforts will not only refine our understanding of *Lycogala* taxonomy and distribution but also contribute significantly to global myxomycete biodiversity assessments.

## Supplementary Material

XML Treatment for Lycogala alisaulianovae

XML Treatment for Lycogala annulatum

XML Treatment for Lycogala caviaroides

XML Treatment for Lycogala chinense

XML Treatment for Lycogala conicum

XML Treatment for Lycogala convexum

XML Treatment for Lycogala epidendrum

XML Treatment for Lycogala exiguum

XML Treatment for Lycogala fasciculovesiculiferum

XML Treatment for Lycogala flavofuscum

XML Treatment for Lycogala fossiculatum

XML Treatment for Lycogala helvolum

XML Treatment for Lycogala indirubinum

XML Treatment for Lycogala irregulare

XML Treatment for Lycogala maculatum

XML Treatment for Lycogala nigrum

XML Treatment for Lycogala planovesiculiferum

XML Treatment for Lycogala projectum

XML Treatment for Lycogala skovorodaense

XML Treatment for Lycogala succineum

XML Treatment for Lycogala uviforme

## ﻿Additional information

### Adherence to national and international regulations

All the specimens used in this study have been legally obtained, respecting the Convention on Biological Diversity (Rio Convention).

### Author ORCIDs

Wen-Long Song https://orcid.org/0000-0002-9573-4108

Zi-Qiong Jiang https://orcid.org/0009-0009-4651-0958

Min Li https://orcid.org/0000-0002-3563-7007

Dmytro Leontyev https://orcid.org/0000-0002-4122-1091

Yang Gao https://orcid.org/0000-0002-7431-274X

Shuang-Lin Chen https://orcid.org/0000-0002-2043-6341

### Data availability

Data will be made available on request.

## Supplementary materials

Supplementary material 1 Details of specimens from GenBank used in the phylogenetic analyses in this study Data type xlsx https://binary.pensoft.net/file/1340394This dataset is made available under the Open Database License (http://opendatacommons.org/licenses/odbl/1.0/). The Open Database License (ODbL) is a license agreement intended to allow users to freely share, modify, and use this Dataset while maintaining this same freedom for others, provided that the original source and author(s) are credited.Wen-Long Song, Zi-Qiong Jiang, Min Li, Dmytro Leontyev, Yang Gao, Shuang-Lin Chen

Supplementary material 2 Full alignment of nuclear 18S rDNA (*SSU*) sequences used for phylogenetic analysis in this study Data type fasta https://binary.pensoft.net/file/1340395This dataset is made available under the Open Database License (http://opendatacommons.org/licenses/odbl/1.0/). The Open Database License (ODbL) is a license agreement intended to allow users to freely share, modify, and use this Dataset while maintaining this same freedom for others, provided that the original source and author(s) are credited.Wen-Long Song, Zi-Qiong Jiang, Min Li, Dmytro Leontyev, Yang Gao, Shuang-Lin Chen

Supplementary material 3 Full alignment of cytochrome oxidase subunit I (* COI*) sequences used for phylogenetic analysis in this study Data type fasta https://binary.pensoft.net/file/1340396This dataset is made available under the Open Database License (http://opendatacommons.org/licenses/odbl/1.0/). The Open Database License (ODbL) is a license agreement intended to allow users to freely share, modify, and use this Dataset while maintaining this same freedom for others, provided that the original source and author(s) are credited.Wen-Long Song, Zi-Qiong Jiang, Min Li, Dmytro Leontyev, Yang Gao, Shuang-Lin Chen

Supplementary material 4 Gblocks-filtered alignment of nuclear 18S rDNA (*SSU*) sequences used for phylogenetic analysis in this study Data type fasta https://binary.pensoft.net/file/1340397This dataset is made available under the Open Database License (http://opendatacommons.org/licenses/odbl/1.0/). The Open Database License (ODbL) is a license agreement intended to allow users to freely share, modify, and use this Dataset while maintaining this same freedom for others, provided that the original source and author(s) are credited.Wen-Long Song, Zi-Qiong Jiang, Min Li, Dmytro Leontyev, Yang Gao, Shuang-Lin Chen

Supplementary material 5 Gblocks-filtered alignment of cytochrome oxidase subunit I (* COI*) sequences used for phylogenetic analysis in this study Data type fasta https://binary.pensoft.net/file/1340398This dataset is made available under the Open Database License (http://opendatacommons.org/licenses/odbl/1.0/). The Open Database License (ODbL) is a license agreement intended to allow users to freely share, modify, and use this Dataset while maintaining this same freedom for others, provided that the original source and author(s) are credited.Wen-Long Song, Zi-Qiong Jiang, Min Li, Dmytro Leontyev, Yang Gao, Shuang-Lin Chen

Supplementary material 6 Maximum likelihood tree inferred from concatenated nuclear 18S rDNA (*SSU*) and cytochrome oxidase subunit I (* COI*) sequences, showing phylogenetic relationships of *Lycogala*. Sale bar indicates substitutions per site Data type pdf https://binary.pensoft.net/file/1340399This dataset is made available under the Open Database License (http://opendatacommons.org/licenses/odbl/1.0/). The Open Database License (ODbL) is a license agreement intended to allow users to freely share, modify, and use this Dataset while maintaining this same freedom for others, provided that the original source and author(s) are credited.Wen-Long Song, Zi-Qiong Jiang, Min Li, Dmytro Leontyev, Yang Gao, Shuang-Lin Chen

Supplementary material 7 ASAP species delimitation results Data type pdf https://binary.pensoft.net/file/1340400This dataset is made available under the Open Database License (http://opendatacommons.org/licenses/odbl/1.0/). The Open Database License (ODbL) is a license agreement intended to allow users to freely share, modify, and use this Dataset while maintaining this same freedom for others, provided that the original source and author(s) are credited.Wen-Long Song, Zi-Qiong Jiang, Min Li, Dmytro Leontyev, Yang Gao, Shuang-Lin Chen

Supplementary material 8 Genetic distance based on nuclear 18S rDNA (*SSU*) sequences Data type html https://binary.pensoft.net/file/1340401This dataset is made available under the Open Database License (http://opendatacommons.org/licenses/odbl/1.0/). The Open Database License (ODbL) is a license agreement intended to allow users to freely share, modify, and use this Dataset while maintaining this same freedom for others, provided that the original source and author(s) are credited.Wen-Long Song, Zi-Qiong Jiang, Min Li, Dmytro Leontyev, Yang Gao, Shuang-Lin Chen
