# Supplementary material for: ﻿Comprehensive revision of Lycogala (Myxomycetes) in subtropical China: morphological and phylogenetic insights and ten new species
Source: IMA Fungus. 2025 May 28;16:e147535. doi: 10.3897/imafungus.16.147535 (PMC12147522; doi:10.3897/imafungus.16.147535)
Supplement: Supplementary material 8 — Genetic distance based on nuclear 18S rDNA (SSU) sequences [file imafungus-16-e147535-s008.html]

ASAP Results 

ASAP Web results  

```
date:2023-05-22T10:11:52
input file:Supplementarymaterial2.fasta
nb of sequences:410
length of seqs:5350
subst. model:K80 Kimura
recurs split pval:1.000000e-02
```

Split probability: 0.010000 sequence length: 5350
  

Save Spart text File  here

Save Spart XML File  here

10 best partitions found by ASAP (see FAQ for more details)   
***The lower the score, the better the partition***

|  |  |  |  |  |  |  |  |  |  |  |  |  |  |  |  |  |  |  |  |  |  |  |  |  |  |  |  |  |  |  |  |  |  |  |  |  |  |  |  |  |  |  |  |  |  |  |  |  |  |  |  |  |  |  |  |  |  |  |  |  |  |  |  |  |  |  |  |  |  |  |  |  |  |  |  |  |  |  |  |  |  |  |
| --- | --- | --- | --- | --- | --- | --- | --- | --- | --- | --- | --- | --- | --- | --- | --- | --- | --- | --- | --- | --- | --- | --- | --- | --- | --- | --- | --- | --- | --- | --- | --- | --- | --- | --- | --- | --- | --- | --- | --- | --- | --- | --- | --- | --- | --- | --- | --- | --- | --- | --- | --- | --- | --- | --- | --- | --- | --- | --- | --- | --- | --- | --- | --- | --- | --- | --- | --- | --- | --- | --- | --- | --- | --- | --- | --- | --- | --- | --- | --- | --- | --- | --- |
| | Nb of subsets | asap-score | P-val (rank) | | W (rank) | Treshold dist. | Text | | --- | --- | --- | --- | --- | --- | --- | | 34 | 3.00 |  | 1.00e-05 (1) | 4.43e-05 (5) | 0.194963 | list csv | | 34 | 5.50 |  | 1.30e-04 (7) | 4.43e-05 (4) | 0.193017 | list csv | | \* 72 | 11.50 |  | 1.09e-03 (9) | 2.77e-05 (14) | 0.028576 | list csv | | 39 | 12.00 |  | 1.00e-04 (6) | 2.68e-05 (18) | 0.151266 | list csv | | \* 78 | 14.50 |  | 2.04e-03 (10) | 2.65e-05 (19) | 0.020825 | list csv | | \* 85 | 15.50 |  | 3.81e-02 (21) | 3.03e-05 (10) | 0.017650 | list csv | | \* 87 | 18.00 |  | 2.90e-04 (8) | 2.27e-05 (28) | 0.015404 | list csv | | \* 71 | 18.50 |  | 1.50e-02 (17) | 2.64e-05 (20) | 0.030014 | list csv | | 39 | 18.50 |  | 1.00e-01 (28) | 3.18e-05 (9) | 0.160097 | list csv | | 42 | 23.50 |  | 6.30e-02 (24) | 2.50e-05 (23) | 0.137093 | list csv |  *Number of subsets in this table equals to the number of primary species hypotheses in the respective partition* | |  |  | | --- | --- | | Histogram of distances [save] | Ranked distances [save] | |  |  | |

  
 **## View/Save Boxed subsets graph here**  


Asap Score
1.0

20.2

39.4

58.6

77.8

97.0

116.2

135.4

154.6

173.8

0.082

0.165

0.247

0.330

0.412

0.495

0.577


dist


Legend:

<0.001

<0.05

<0.1

>0.1

N/A


Lycogala\_mac

Lycogala\_mac

Lycogala\_epi

Lycogala\_epi

Lycogala\_epi

Lycogala\_epi

Lycogala\_sp.

Lycogala\_sp.

Lycogala\_sp.

HFNNU\_11333

Lycogala\_sp.

Lycogala\_sp.

Lycogala\_epi

Lycogala\_epi

Lycogala\_epi

Lycogala\_epi

Lycogala\_epi

Lycogala\_epi

Lycogala\_epi

Lycogala\_epi

Lycogala\_epi

Lycogala\_epi

Lycogala\_epi

Lycogala\_epi

Lycogala\_epi

HFNNU\_11266

HFNNU\_11267

Lycogala\_epi

HFNNU\_11239

HFNNU\_11240

HFNNU\_11241

HFNNU\_11242

Lycogala\_epi

Lycogala\_epi

Lycogala\_epi

Lycogala\_mac

Lycogala\_fos

Lycogala\_epi

Lycogala\_epi

Lycogala\_epi

Lycogala\_epi

HFNNU\_11236

HFNNU\_11237

HFNNU\_11238

Lycogala\_epi

Lycogala\_epi

Lycogala\_epi

Lycogala\_epi

Lycogala\_epi

Lycogala\_epi

Lycogala\_epi

Lycogala\_mac

Lycogala\_epi

Lycogala\_epi

Lycogala\_suc

Lycogala\_suc

Lycogala\_suc

Lycogala\_suc

Lycogala\_suc

Lycogala\_suc

Lycogala\_sko

Lycogala\_epi

Lycogala\_epi

Lycogala\_epi

Lycogala\_epi

HFNNU\_11270

HFNNU\_11282

Lycogala\_mac

Lycogala\_epi

HFNNU\_11247

HFNNU\_11248

HFNNU\_11249

Lycogala\_epi

Lycogala\_epi

Lycogala\_epi

Lycogala\_epi

Lycogala\_epi

Lycogala\_epi

Lycogala\_epi

Lycogala\_epi

Lycogala\_epi

Lycogala\_mac

Lycogala\_mac

Lycogala\_epi

HFNNU\_11243

HFNNU\_11244

HFNNU\_11245

HFNNU\_11246

Lycogala\_sp.

Lycogala\_epi

Lycogala\_epi

Lycogala\_epi

Lycogala\_epi

Lycogala\_epi

Lycogala\_fos

Lycogala\_epi

Lycogala\_epi

Lycogala\_epi

Lycogala\_epi

Lycogala\_mac

Lycogala\_mac

Lycogala\_mac

Lycogala\_mac

Lycogala\_epi

Lycogala\_epi

Lycogala\_epi

Lycogala\_epi

Lycogala\_gut

Lycogala\_gut

Lycogala\_epi

Lycogala\_epi

Lycogala\_epi

Lycogala\_epi

Lycogala\_epi

Lycogala\_sp.

Lycogala\_sp.

Lycogala\_sp.

HFNNU 10821

HFNNU 10819

HFNNU\_10820

HFNNU\_11346

HFNNU\_10824

HFNNU\_10823

HFNNU\_10825

HFNNU\_11344

HFNNU\_11345

HFNNU\_10829

HFNNU\_10826

HFNNU\_10827

Lycogala\_gut

Lycogala\_gut

Lycogala\_sko

Lycogala\_sko

Lycogala\_sko

Lycogala\_fos

HFNNU\_10835

HFNNU\_10836

Lycogala\_fos

Lycogala\_fos

Lycogala\_fos

Lycogala\_fos

Lycogala\_ali

Lycogala\_irr

Lycogala\_irr

Lycogala\_irr

Lycogala\_irr

Lycogala\_agg

Lycogala\_agg

Lycogala\_ali

Lycogala\_ali

Lycogala\_ali

Lycogala\_ali

Lycogala\_ali

Lycogala\_con

Lycogala\_con

Lycogala\_con

Lycogala\_con

Lycogala\_con

Lycogala\_con

Lycogala\_con

Lycogala\_con

Lycogala\_agg

Lycogala\_bot

Lycogala\_bot

Lycogala\_bot

Lycogala\_bot

Lycogala\_con

Lycogala\_exi

Lycogala\_exi

Lycogala\_fos

Lycogala\_irr

Lycogala\_irr

Lycogala\_leo

Lycogala\_mac

Lycogala\_ole

Lycogala\_ole

Lycogala\_onc

Lycogala\_onc

Lycogala\_onc

Lycogala\_pal

Lycogala\_pal

Lycogala\_sko

Lycogala\_sko

Lycogala\_sko

Lycogala\_sko

Lycogala\_sko

Lycogala\_con

Lycogala\_fla

Lycogala\_aci

Lycogala\_aci

Lycogala\_ali

Lycogala\_cav

Lycogala\_cav

Lycogala\_epi

Lycogala\_epi

Lycogala\_epi

Lycogala\_epi

Lycogala\_epi

Lycogala\_epi

Lycogala\_epi

Lycogala\_epi

Lycogala\_epi

Lycogala\_epi

Lycogala\_ros

Lycogala\_ros

Lycogala\_ros

Lycogala\_het

Lycogala\_epi

Lycogala\_epi

Lycogala\_suc

Lycogala\_suc

Lycogala\_suc

Lycogala\_suc

Lycogala\_suc

Lycogala\_suc

Lycogala\_suc

Lycogala\_suc

Lycogala\_suc

Lycogala\_suc

Lycogala\_suc

Lycogala\_ole

Lycogala\_ole

Lycogala\_ole

Lycogala\_ole

Lycogala\_ole

Lycogala\_ole

Lycogala\_ole

Lycogala\_ole

Lycogala\_ole

Lycogala\_ole

Lycogala\_ole

Lycogala\_ole

Lycogala\_con

Lycogala\_con

Lycogala\_con

Lycogala\_con

Lycogala\_con

Lycogala\_con

Lycogala\_con

Lycogala\_con

Lycogala\_con

Lycogala\_fla

Lycogala\_fla

Lycogala\_fla

Lycogala \_ma

Lycogala \_ma

Lycogala \_ma

Lycogala \_ma

Lycogala \_ma

Lycogala \_ma

Lycogala \_ma

Lycogala \_ma

Lycogala \_ma

Lycogala \_ma

Lycogala \_ma

Lycogala\_pal

Lycogala\_pal

Lycogala\_pal

Lycogala\_pal

Lycogala\_"ru

Lycogala\_"ru

Lycogala\_"gu

Lycogala\_leo

Lycogala\_leo

Lycogala\_leo

Lycogala\_leo

Lycogala\_leo

Lycogala\_leo

Lycogala\_leo

Lycogala\_leo

Lycogala\_leo

Lycogala\_aci

Lycogala\_aci

Lycogala\_aci

Lycogala\_aci

Lycogala\_epi

Lycogala\_epi

Lycogala\_epi

Lycogala\_epi

Lycogala\_mac

Lycogala\_mac

Lycogala\_irr

Lycogala\_irr

Lycogala\_irr

Lycogala\_irr

Lycogala\_irr

Lycogala\_irr

Lycogala\_epi

Lycogala\_epi

Lycogala\_sp.

Lycogala\_sp.

Lycogala\_sp.

Lycogala\_epi

Lycogala\_epi

Lycogala\_epi

Lycogala\_"mi

Lycogala\_"mi

Lycogala\_epi

Lycogala\_epi

Lycogala\_cav

Lycogala\_cav

Lycogala\_cav

Lycogala\_epi

Lycogala\_epi

Lycogala\_epi

Lycogala\_epi

Lycogala\_epi

Lycogala\_epi

Lycogala\_epi

Lycogala\_sp\_

Lycogala\_epi

Lycogala\_epi

Lycogala\_epi

Lycogala\_epi

Lycogala\_epi

Lycogala\_epi

Lycogala\_epi

Lycogala\_sp\_

Lycogala\_sp\_

Lycogala\_sp\_

Lycogala\_"ol

Lycogala\_"me

Lycogala\_epi

Lycogala\_epi

Lycogala\_epi

Lycogala\_epi

Lycogala\_epi

Lycogala\_epi

Lycogala\_epi

Lycogala\_epi

Lycogala\_epi

Lycogala\_epi

Lycogala\_epi

Lycogala\_epi

Lycogala\_epi

Lycogala\_epi

Lycogala\_epi

Lycogala\_epi

Lycogala\_epi

Lycogala\_epi

Lycogala\_epi

Lycogala\_epi

Lycogala\_epi

Lycogala\_epi

Lycogala\_epi

Lycogala\_epi

Lycogala\_epi

Lycogala\_epi

Lycogala\_epi

Lycogala\_epi

Lycogala\_epi

Lycogala\_epi

Lycogala\_epi

Lycogala\_epi

Lycogala\_epi

Lycogala\_epi

Lycogala\_epi

Lycogala\_epi

Lycogala\_epi

Lycogala\_epi

Lycogala\_epi

Lycogala\_epi

Lycogala\_epi

Lycogala\_epi

Lycogala\_con

Lycogala\_con

Lycogala\_ali

Lycogala\_ali

Lycogala\_con

Lycogala\_fla

Lycogala\_fla

Lycogala\_irr

Lycogala\_irr

Lycogala\_irr

Lycogala\_irr

Lycogala\_leo

Lycogala\_leo

Lycogala\_leo

Lycogala\_ole

Lycogala\_ole

Lycogala\_pal

Lycogala\_pal

Lycogala\_pal

Lycogala\_pal

Lycogala\_pal

Lycogala\_suc

Lycogala\_epi

Lycogala\_epi

Lycogala\_epi

Lycogala\_epi

Lycogala\_epi

Lycogala\_epi

Lycogala\_"ol

HFNNU\_11283

HFNNU\_11284

HFNNU\_11285

HFNNU\_11286

HFNNU\_11287

HFNNU\_11288

HFNNU\_11289

TNSM13641\_as

Lycogala\_sp.

Lycogala\_sp.

Lycogala\_sp.

Lycogala\_sp.

Lycogala\_sp.

Lycogala\_sp.

Lycogala\_sp.

Lycogala\_sp.

Lycogala\_sp.


Tooltip
  
 View/save curves and dendrogram here  
  
*Responsive Crossing lines Legend: Green Line= grouping distance (Dc)- Red line = treshold distance(Dt)*  
Running time:
4 min 15 seconds
  
