# Supplementary material for: ﻿Comprehensive revision of Lycogala (Myxomycetes) in subtropical China: morphological and phylogenetic insights and ten new species
Source: IMA Fungus. 2025 May 28;16:e147535. doi: 10.3897/imafungus.16.147535 (PMC12147522; doi:10.3897/imafungus.16.147535)

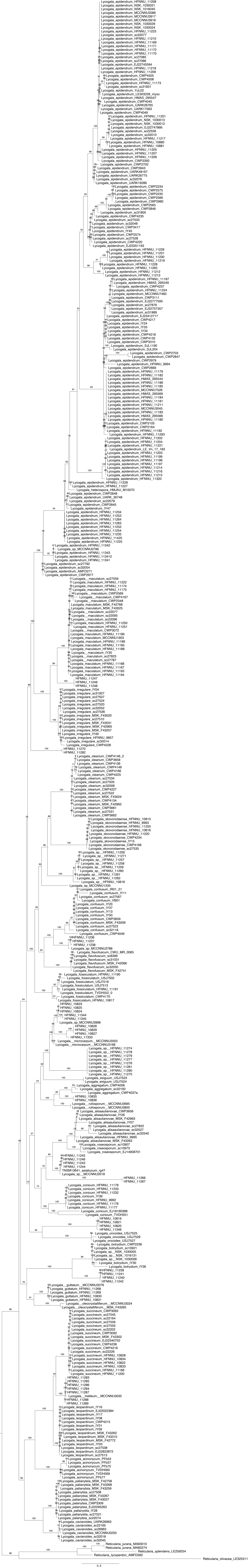

Supplement: Supplementary material 6 — Maximum likelihood tree inferred from concatenated nuclear 18S rDNA (SSU) and cytochrome oxidase subunit I ( COI) sequences, showing phylogenetic relationships of Lycogala. Sale bar indicates substitutions per site [file imafungus-16-e147535-s006.pdf]
